# Identification of Differential Drought Response Mechanisms in *Medicago sativa* subsp. *sativa* and *falcata* through Comparative Assessments at the Physiological, Biochemical, and Transcriptional Levels

**DOI:** 10.3390/plants10102107

**Published:** 2021-10-05

**Authors:** Stacy D. Singer, Udaya Subedi, Madeline Lehmann, Kimberley Burton Hughes, Biruk A. Feyissa, Abdelali Hannoufa, Bin Shan, Guanqun Chen, Kazi Kader, Rodrigo Ortega Polo, Timothy Schwinghamer, Gaganpreet Kaur Dhariwal, Surya Acharya

**Affiliations:** 1 Lethbridge Research and Development Centre, Agriculture and Agri-Food Canada, Lethbridge, AB T1J 4B1, Canada; udaya.subedi@agr.gc.ca (U.S.); madeline.lehmann@agr.gc.ca (M.L.); kimberley.burtonhughes@agr.gc.ca (K.B.H.); kakader35@yahoo.com (K.K.); rodrigo.ortegapolo@agr.gc.ca (R.O.P.); timothy.schwinghamer@agr.gc.ca (T.S.); gaganpreet.dhariwal@agr.gc.ca (G.K.D.); surya.acharya@agr.gc.ca (S.A.); 2 Department of Agricultural, Food and Nutritional Science, University of Alberta, Edmonton, AB T6G 2P5, Canada; bshan@ualberta.ca (B.S.); gc24@ualberta.ca (G.C.); 3 London Research and Development Centre, Agriculture and Agri-Food Canada, London, ON N5V 4T3, Canada; bfeyissa@uwo.ca (B.A.F.); Abdelali.Hannoufa@agr.gc.ca (A.H.)

**Keywords:** alfalfa, differential gene expression, drought tolerance, forage, stress response

## Abstract

Alfalfa (*Medicago sativa* L.) is an extensively grown perennial forage legume, and although it is relatively drought tolerant, it consumes high amounts of water and depends upon irrigation in many regions. Given the progressive decline in water available for irrigation, as well as an escalation in climate change-related droughts, there is a critical need to develop alfalfa cultivars with improved drought resilience. *M. sativa* subsp. *falcata* is a close relative of the predominantly cultivated *M. sativa* subsp. *sativa*, and certain accessions have been demonstrated to exhibit superior performance under drought. As such, we endeavoured to carry out comparative physiological, biochemical, and transcriptomic evaluations of an as of yet unstudied drought-tolerant *M. sativa* subsp. *falcata* accession (PI 641381) and a relatively drought-susceptible *M. sativa* subsp. *sativa* cultivar (Beaver) to increase our understanding of the molecular mechanisms behind the enhanced ability of *falcata* to withstand water deficiency. Our findings indicate that unlike the small number of *falcata* genotypes assessed previously, *falcata* PI 641381 may exploit smaller, thicker leaves, as well as an increase in the baseline transcriptional levels of genes encoding particular transcription factors, protective proteins, and enzymes involved in the biosynthesis of stress-related compounds. These findings imply that different *falcata* accessions/genotypes may employ distinct drought response mechanisms, and the study provides a suite of candidate genes to facilitate the breeding of alfalfa with enhanced drought resilience in the future.

## 1. Introduction

Alfalfa (*Medicago sativa* L.) is one of the most widely grown and valuable perennial forage legumes, with an estimated global cropping area of over 30 million hectares. This popularity stems from the high nutritional value, palatability, environmental adaptability, and biomass yield of alfalfa, as well as its low fertilizer requirements due to its ability to fix nitrogen through symbiosis with rhizobia [1]. The demand for ruminant products such as meat and milk is expected to grow substantially in coming years due to our expanding population and affluence, and high levels of forage crop production will therefore be a necessity [2]. While alfalfa is relatively drought tolerant compared to many other crop species as a result of the typical presence of a deep tap root, its production depends upon irrigation in many growing regions, and it consumes a particularly high amount of water due to its long growing season and dense canopy [3]. Unfortunately, there is a progressively limited supply of water for irrigation [4], as well as an escalation in the severity and frequency of drought events due to climate change [5], which will negatively impact alfalfa productivity [6]. As such, there is a vital need to develop alfalfa cultivars that use water more efficiently and/or exhibit improved drought resilience compared to current varieties in a timely manner [7,8]. 

Plants respond to drought stress using a variety of physiological, cellular, and molecular processes, with the aim of enhancing their ability to withstand or recover from such challenging conditions. At the molecular level, drought stress leads to an increase in the production of reactive oxygen species (ROS) that act as important signaling molecules in the abiotic stress response. However, when present at levels above a particular threshold, they can be detrimental to plant cells due to resulting lipid peroxidation, as well as the degradation of nucleic acids and proteins [9]. In order to minimize such damage, plants tend to enhance the production/activity of various enzymatic and non-enzymatic antioxidants under water deficit as a means of scavenging ROS and maintaining redox homeostasis [10]. ROS can also influence the production of certain phytohormones, which then often function through an interplay with the ROS signaling cascade [11]. Abscisic acid (ABA) in particular is known to be a key signaling hormone under drought stress in plants, where its accumulation leads to stomatal closure and the regulation of numerous transcription factors [12]. The differential activity of these ABA-responsive transcription factors, as well as the transcriptional regulation of many genes that are modulated in ABA-independent pathways, then leads to an increased accumulation of various products that contribute to stress tolerance, including protective proteins such as late embryogenesis abundant (LEA) proteins, osmoprotectants such as proline, glycine betaine, and trehalose, and the aforementioned antioxidant system components [13]. Although this general framework for the cascade of events that occurs following plant exposure to drought stress has been elucidated, the complex regulatory processes that coordinate these responses have yet to be established in full at the genetic level, and precise mechanisms can differ between plant species and genotypes. Above and beyond drought tolerance mechanisms, plants can also make use of drought avoidance, which typically entails a short life cycle and/or developmental plasticity, and drought escape, which tends to involve enhanced water uptake and/or reduced water loss [13].

Until fairly recently, the enhancement of drought resilience has not been a major focus of alfalfa breeding efforts, and the scope of genetic variation and mechanisms responsible for this trait are not fully known [14]. While conventional breeding programs have begun to focus on the improvement of this trait in alfalfa [2,15], and a growing number of studies are dedicated towards the enhancement of drought tolerance in alfalfa using a transgenic approach [16,17,18,19,20], such efforts are complicated by a lack of understanding of the exact mechanisms by which alfalfa senses, reacts, and adapts to water deficit. *Medicago sativa* subsp. *falcata*, which can be found in diploid or tetraploid form, is a close relative of tetraploid *M. sativa* subsp. *sativa*. While the *sativa* subspecies is the most commonly cultivated subspecies of alfalfa, current-day varieties have been bred using introgressions from, and hybridization with, other subspecies, including *falcata*, and thus the delineation between subspecies is not necessarily quite so obvious [2]. In any case, *M. sativa* subsp. *falcata* is well known to exhibit superior cold tolerance compared to the *sativa* subspecies [21,22], and a small number of studies have also shown it to be more resilient to other abiotic stresses such as drought [22,23,24,25]. While *M. sativa* subsp. *falcata* germplasm has been used for many years in alfalfa breeding programs as a means of harnessing its enhanced abiotic stress tolerance, it typically exhibits relatively low productivity and persistence [15,26,27], which has made its use in this context challenging. 

The drought resilience of the *falcata* subspecies has been suggested to result from several physiological and biochemical characteristics, including reduced stomatal density and conductance, delayed leaf senescence, increased root length, and a greater accumulation of carbohydrate osmoprotectants (raffinose, myo-inositol, and galactinol) and flavonoid antioxidants [24,25]. In addition, various studies have demonstrated that the heterologous expression of certain genes from *M. sativa* subsp. *falcata* genotypes, including those encoding Universal Stress Protein 1 (*MfUSP1*) [19], galactinol synthase (*MfGolS1*) [16], *myo*-inositol phosphate synthase (*MfMIPS1*) [28], and late embryogenesis abundant protein 3 (*MfLEA3*) [20], in tobacco led to enhanced tolerance to osmotic or dehydration stress in transgenic lines. Although comparative transcriptional responses between a drought-sensitive *sativa* (Chilean) and a drought-tolerant *falcata* (Wisfal) variety have been assessed under drought stress using microarray analysis previously [24], this type of evaluation has not been extended to other accessions/genotypes as of yet. Therefore, we sought to carry out further physiological, biochemical, and transcriptomic comparisons of distinct *falcata* and *sativa* accessions exhibiting differential levels of drought tolerance to provide new insight into the molecular mechanisms driving the resilience of *falcata* to water-deficient environmental conditions. 

## 2. Results

### 2.1. The ‘Falcata’ Genotype Exhibits an Enhanced Ability to Withstand Drought Conditions Compared to ‘Sativa’

**Figure 1:**
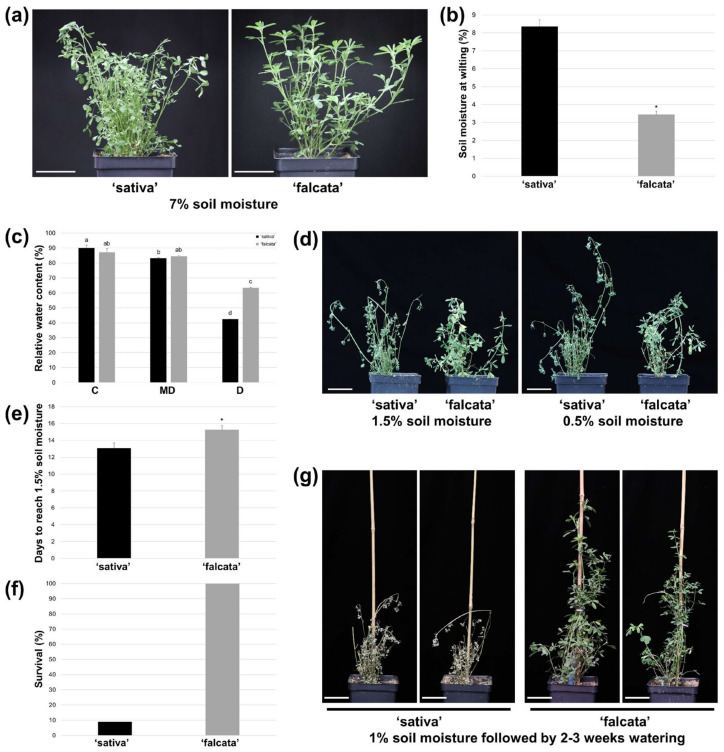
Response of ‘sativa’ and ‘falcata’ genotypes to drought stress conditions. (**a**) Representative images of ‘sativa’ and ‘falcata’ after withholding water until volumetric soil moisture levels reached approximately 7%. (**b**) Soil moisture level at which ‘sativa’ and ‘falcata’ began to wilt. (**c**) Relative water content (RWC) of first fully expanded trifoliate leaves assessed when soil moisture contents were at ~50% (C, control), ~20% (MD, mild drought), and ~7% (D, drought). (**d**) Representative images of ‘sativa’ and ‘falcata’ after withholding water until soil moisture levels reached 1.5% and 0.5%, respectively. (**e**) Number of days from the initiation of drought treatment at which soil moisture levels in pots reached approximately 1.5%. (**f**) Proportion of plants that survived following 2–3 weeks of re-watering after allowing soil moisture contents to reach 1%. (**g**) Representative images of ‘sativa’ and ‘falcata’ plants following 2–3 weeks of re-watering once plants reached 1% soil moisture content. Blocks in each graph consist of the mean value of 10 (**e**) or 11 (**b**,**c**,**f**) biological replicates derived from vegetative stem cuttings, with black denoting ‘sativa’ and gray denoting ‘falcata’. Bars denote standard errors. Scale bars = 5 cm. Lowercase letters indicate statistically significant differences between groups in each graph (*p* ≤ 0.05), while asterisks denote means that are statistically different in ‘falcata’ compared to ‘sativa’ (*p* ≤ 0.01).

To assess the extent of differential drought responses between *M. sativa* subsp. *falcata* accession PI 641381 (hereafter referred to as ‘falcata’) and *sativa* cultivar Beaver (hereafter referred to as ‘sativa’), water was withheld from plants, volumetric soil moisture contents were measured daily, and symptoms of stress were monitored. ‘Sativa’ plants typically began to wilt when volumetric soil moisture levels reached between 7–9%, while ‘falcata’ plants did not exhibit any signs of drought stress at this point (Figure 1a,b) and instead only began to show symptoms at between 3% and 4% soil moisture levels, which was significantly lower than ‘sativa’ (Figure 1b). In line with this, while leaf relative water content (RWC) declined significantly in both ‘sativa’ and ‘falcata’ genotypes under drought conditions (soil moisture level of approximately 7%) compared to well-watered conditions, only ‘sativa’ exhibited a significant reduction in RWC under moderate drought conditions (soil moisture level of approximately 20%) compared to control growth conditions (Figure 1c). In addition, RWC was significantly higher in ‘falcata’ than ‘sativa’ under more severe drought conditions (soil moisture level of 7%; Figure 1c). Similarly, ‘falcata’ plants also consistently exhibited fewer detrimental effects from severe drought stress than ‘sativa’, and even at soil moisture levels of 0.5–1.5%, when ‘sativa’ plants were severely desiccated, ‘falcata’ plants retained some level of turgidity (Figure 1d). We also evaluated the number of days from the commencement of drought treatment for soil moisture levels to reach approximately 1.5%, and found that ‘falcata’ took significantly longer to reach a low soil moisture level than ‘sativa’ (Figure 1e). Finally, subsequent to having re-watered plants for 2–3 weeks after they reached a soil moisture level of approximately 1%, only 10% of ‘sativa’ plants survived, while 100% of ‘falcata’ plants regenerated (Figure 1f,g). Taken together, these results suggest that not only is the ‘falcata’ genotype more resilient to drought stress than ‘sativa’, but that this genotype also may use water at a lower rate.

### 2.2. ‘Falcata’ Plants Exhibit Differences in Growth Characteristics Compared to ‘Sativa’ under Well-Watered Conditions and Following Drought Recovery

**Figure 2:**
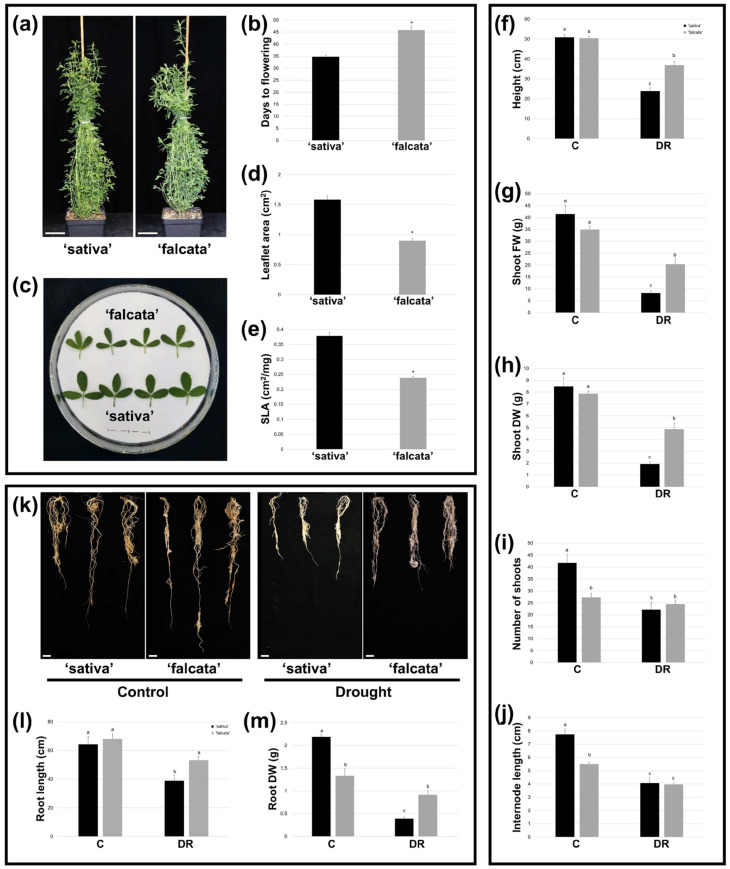
Aboveground and belowground morphological characteristics of ‘sativa’ and ‘falcata’ genotypes grown under well-watered conditions, as well as following drought recovery. (**a**) Representative images of ‘sativa’ and ‘falcata’ taken approximately 1 month following cutting. Scale bars = 5 cm. (**b**) Number of days following cutting until plants flowered under well-watered conditions. (**c**) Representative image of ‘sativa’ and ‘falcata’ trifoliate leaves (third from shoot tip) under well-watered conditions. Scale bar = 4 cm. (**d**) Area of middle leaflet of third fully expanded trifoliate leaf from shoot tip under well-watered conditions. (**e**) Specific leaf area (SLA) of middle leaflet of third trifoliate leaf from shoot tip under well-watered conditions. (**f**–**j**) Length of the longest shoot, aboveground fresh weight (FW) and dry weight (DW), total number of shoots, and length of the longest internode in ‘sativa’ and ‘falcata’ plants under control conditions (C, approximately 50% soil moisture content) and following drought recovery (DR, 2 weeks of re-watering after allowing soil moisture levels to reach approximately 4%). Measurements were taken 35 (**f**–**h**) or 37 (**i**,**j**) days following cutting. (**k**) Representative images of ‘sativa’ and ‘falcata’ roots 54 days following cutting under control conditions (approximately 50% soil moisture content) and following drought recovery (3 weeks of re-watering after allowing soil moisture levels to reach approximately 1%). Scale bars = 5 cm. (**l**–**m**) Length of the longest root and root DW in ‘sativa’ and ‘falcata’ plants under control conditions (C, approximately 50% soil moisture content) and following drought recovery (DR, approximately 3 weeks of re-watering after allowing soil moisture levels to reach approximately 1% soil moisture content). Measurements were taken 54 days following cutting. For all graphs, blocks consist of the mean value of 5 (**d**,**e**) or 11 (**f**–**j**,**l**–**m**) biological replicates derived from stem cuttings, with black denoting ‘sativa’ and gray denoting ‘falcata’. Bars denote standard errors. Lowercase letters indicate statistically significant differences between groups in each graph (*p* ≤ 0.05), while asterisks denote means that are statistically different in ‘falcata’ compared to ‘sativa’ (*p* ≤ 0.01).

To determine whether ‘sativa’ and ‘falcata’ genotypes exhibited differential growth responses, plant growth characteristics were first assessed under well-watered conditions (approximately 50% soil moisture content). Under such non-limiting conditions, ‘sativa’ and ‘falcata’ plants appeared morphologically similar overall (Figure 2a); however, quantitative differences were apparent between the two genotypes. Flowering was significantly delayed in ‘falcata’ compared to ‘sativa’ under well-watered conditions (Figure 2b), with the first open flowers noted in ‘sativa’ and ‘falcata’ on average 35 and 45 days following cutting, respectively. Leaf size was also substantially smaller in ‘falcata’ than ‘sativa’ (Figure 2c), with a significant reduction in leaflet area (43.3% relative decrease compared to ‘sativa’; Figure 2d) and specific leaf area (SLA; 37.0% relative decrease compared to ‘sativa’; Figure 2e) compared to ‘sativa’ under control growth conditions. While no significant differences were noted between genotypes in terms of plant height (Figure 2f) or aboveground biomass (Figure 2g,h) under well-watered conditions, the ‘falcata’ genotype exhibited significantly fewer shoots (Figure 2i) and reduced internode length (Figure 2j). In terms of root characteristics, there was no significant difference in root length in ‘falcata’ compared to ‘sativa’ under control conditions (Figure 2k,l); however, a significant decrease in root dry weight (DW) was noted in ‘falcata’ compared to ‘sativa’ (Figure 2m).

Plant growth characteristics were also assessed following drought recovery (2–3 weeks of re-watering subsequent to drought treatment) as a means of distinguishing possible differences in biomass yield penalties between genotypes. Both ‘falcata’ and ‘sativa’ displayed significant reductions in plant height (Figure 2f), internode length (Figure 2j), and aboveground fresh weight (FW) and DW biomass (Figure 2g,h) after recovery from drought treatment. However, reductions were less substantial in ‘falcata’ than ‘sativa’ in every case. Similarly, while ‘sativa’ exhibited a significant reduction in shoot number following drought recovery compared to plants that had been well-watered, no statistically significant change was noted in ‘falcata’ between conditions (Figure 2i). In terms of root growth, while significant decreases in both root length (39.4% relative decrease) and root DW (82.0% relative decrease) were observed in ‘sativa’ plants that were re-watered following drought treatment compared to unstressed plants, no significant differences were observed in ‘falcata’ plants between treatments for either trait. This corresponded with significant increases in both root length and DW in ‘falcata’ compared to ‘sativa’ following re-watering (Figure 2l,m). Taken together, these results suggest that shoot and root growth penalties were less severe following drought in ‘falcata’ than ‘sativa’. 

### 2.3. Stomatal Density and Size, but Not Photosynthesis-Related Traits or Detached Leaf Water Loss, Differ between ‘Sativa’ and ‘Falcata’ Genotypes

To gain further insight into the physiological differences between the ‘sativa’ and ‘falcata’ genotypes assessed in this study, stomatal- and photosynthesis-related traits were evaluated, and detached leaf water loss assays were carried out. No significant differences were found between ‘sativa’ and ‘falcata’ in detached leaf water loss assays at any time point (Figure S1a), or between genotypes or growth conditions (approximately 50% (control) and 8% (drought) soil moisture contents) with respect to leaf chlorophyll content (Figure S1b). In terms of stomatal traits under well-watered conditions, the ‘falcata’ genotype exhibited a significant 40.2% relative increase in abaxial stomatal density compared to ‘sativa’ (Figure S1c,d). Conversely, while no significant difference in stomatal length was noted between genotypes under well-watered conditions in this study (Figure S1e), stomatal width and area were significantly reduced in ‘falcata’ compared to ‘sativa’ (15.8% and 17.7% relative decreases, respectively; Figure S1f,g). 

Light-saturated photosynthetic rate (Asat), stomatal conductance (*g*_s_), and transpiration rate (E) were all found to decrease significantly under drought conditions (volumetric soil moisture content of approximately 7%) compared to well-watered conditions in both ‘sativa’ and ‘falcata’. However, no significant differences were noted between genotypes (Figure S1h–j), which indicates that these traits were not a main driver for the observed differences in drought tolerance.

### 2.4. ‘Sativa’ and ‘falcata’ Genotypes Exhibit Differential Soluble Carbohydrate Levels and Antioxidant Activities

Levels of osmoprotectants, including proline and soluble carbohydrates, and antioxidant activities were assessed in leaf tissues from plants grown under well-watered and drought conditions (volumetric soil moisture contents of approximately 7%) to gain a further understanding of possible mechanisms driving the superior drought tolerance observed in ‘falcata’ compared to ‘sativa’ in this study. Proline concentrations increased significantly under drought compared to control conditions in both genotypes (25.4% and 19.9% relative increases in ‘sativa’ and ‘falcata’, respectively); however, no significant differences were noted between genotypes under either growth condition (Figure S2a). Both ‘sativa’ and ‘falcata’ also exhibited significant enhancements in soluble carbohydrate levels under drought compared to the control treatment (47.2% and 47.0% relative increases in ‘sativa’ and ‘falcata’, respectively); however, in this case, levels were significantly lower in ‘falcata’ than ‘sativa’ under both growth conditions (Figure S2b). 

In terms of antioxidant activity, superoxide dismutase (SOD) activity was found to be significantly lower in ‘falcata’ than ‘sativa’ under both well-watered and drought conditions (Figure S2c). Conversely, no significant differences in ascorbate peroxidase (APX) activity were noted between growth conditions or genotypes in this study (Figure S2d). In the case of catalase (CAT) activity, while no significant differences were observed between genotypes under well-watered or drought conditions, ‘sativa’, but not ‘falcata’, exhibited a significant increase in activity under drought conditions compared to control conditions (36.4% relative increase; Figure S2e).

### 2.5. RNA-Seq Analysis of ‘Sativa’ and ‘Falcata’ Leaf Tissue under Control and Drought Conditions

To determine whether the ‘sativa’ and ‘falcata’ accessions assessed here exhibited differential gene expression under control and drought conditions, we carried out comparative RNA-Seq analysis using RNA derived from the leaves of a ‘sativa’ and ‘falcata’ genotype, respectively, under both well-watered and drought conditions (at an average volumetric soil moisture content of approximately 7%; four biological replicates of each) for a total of 16 samples. Between 79,873,614 and 178,252,170 reads, with an average of 100,797,720 reads, were obtained per sample, leading to a mean coverage of 20,361,139,390 bases per sample (Table S1). The resulting reads were mapped to the *M. truncatula* reference genome (v4.0), with an average of approximately 36,074 and 36,716 transcripts identified under well-watered conditions in ‘sativa’ and ‘falcata’, respectively, and 38,149 and 38,041 transcripts identified under drought in ‘sativa’ and ‘falcata’, respectively (Table S1). Two of the test groups (‘sativa’ control and ‘falcata’ drought) formed distinct groups after principal component analysis (PCA) of fragments per kilobase of transcript per million mapped reads (FPKM), which confirmed the similarity of biological replicates within each group. Conversely, ‘sativa’ drought and ‘falcata’ control groups both included three biological replicates that formed distinct groups following PCA analysis, while one biological replicate of each was outlying to some degree (Figure S3). Some variability among biological replicates may be expected due to minor differences in microclimate or developmental stage, and overall there was a clear separation among groups of samples in this study.

We first aimed to assess transcriptional discrepancies in both ‘sativa’ and ‘falcata’ genotypes, respectively, between control and drought conditions (File S1). A comparison between ‘sativa’ plants grown under well-watered and drought conditions led to the identification of 6,520 differentially expressed genes (DEGs), with 3104 exhibiting significant up-regulation under drought conditions compared to control conditions, and 3416 DEGs displaying significant down-regulation under drought. Similarly, a comparison of ‘falcata’ plants under well-watered and drought conditions led to the identification of 4354 DEGs, with 1574 DEGs exhibiting significant up-regulation under drought compared to control conditions, and 2780 DEGs displaying significant down-regulation under drought. Of these, 3494 DEGs were unique to ‘sativa’, 1328 DEGs were unique to ‘falcata’, and 3026 DEGs were present in both ‘sativa’ and ‘falcata’ between control vs. drought conditions (Figure S4a). These results suggest that the more drought-sensitive ‘sativa’ genotype was affected to a greater degree transcriptionally than the drought-tolerant ‘falcata’ genotype under drought stress compared to control conditions. 

We also assessed differential gene expression between ‘sativa’ and ‘falcata’ genotypes under both control and drought conditions (File S1). A cross-comparison of ‘sativa’ and ‘falcata’ plants grown under well-watered conditions led to the identification of 490 DEGs between the two subspecies, with 373 genes displaying significantly increased transcript abundance, and 117 exhibiting significantly decreased transcript abundance in ‘falcata’ compared to ‘sativa’. Conversely, a comparison between ‘sativa’ and ‘falcata’ plants grown under drought conditions led to the identification of 4304 DEGs, with 1991 being significantly up-regulated and 2313 being significantly down-regulated in ‘falcata’ compared to ‘sativa’. Of these, 238 DEGs were specific to control conditions, 4052 were specific to drought conditions, and 252 were present under both control and drought conditions (Figure S4b). These results indicate that greater transcriptional differences were apparent between ‘sativa’ and ‘falcata’ under drought stress than under control conditions. 

Comparison of quantitative real-time RT-PCR (qRT-PCR) and RNA-Seq log2 fold-change values for ten genes identified as DEGs in our RNA-Seq analyses revealed a high level of correlation between the results (Figure S4c,d). Correlation coefficients of 0.99 and 0.97 were observed across RNA-Seq and qRT-PCR log2 fold-change values between control and drought conditions in ‘sativa’ and ‘falcata’ genotypes for these ten genes, which confirms the validity of the RNA-Seq data in this study.

### 2.6. Differential Gene Ontology (GO) Term Enrichment in ‘Sativa’ and ‘Falcata’ under Drought Compared to Control Conditions 

In order to assess ‘sativa’ and ‘falcata’ in terms of their transcriptional response to drought, we carried out singular enrichment analysis (SEA) and subsequent cross comparison of SEA (SEACOMPARE) of both ‘sativa’ and ‘falcata’ DEGs, respectively, that were observed under drought compared to control conditions. For genes that were up-regulated under drought compared to well-watered conditions in the biological process GO term category, no significantly enriched terms were observed in ‘sativa’, while 33 terms were significantly enriched in ‘falcata’ (Figure S5). In the molecular function GO term category, significant enrichment of 13 and 7 GO terms were observed in ‘sativa’ and ‘falcata’ genotypes, respectively (Figure S6). No significantly enriched GO terms derived from genes that were up-regulated in drought vs. control conditions were identified in either genotype in the cellular component category.

For genes that were down-regulated in drought compared to well-watered conditions, far more GO terms were enriched in ‘sativa’ and ‘falcata’ in the biological process, molecular function, and cellular compartment categories than for up-regulated genes. In addition, substantially more GO terms were enriched overall for down-regulated genes in ‘sativa’ than ‘falcata’. In the case of the biological process category, 102 GO terms were significantly enriched in ‘sativa’, whereas only 43 were identified in ‘falcata’. While 39 terms were enriched in both ‘sativa’ and ‘falcata’, enrichment was typically more significant in ‘sativa’ than ‘falcata’ (Figure S7). In the molecular function category, 25 significantly enriched GO terms were identified in ‘sativa’ and ‘falcata’ genotypes. Of these, 11 were significantly enriched in ‘sativa’ but not ‘falcata’, while 8 were significantly enriched in ‘falcata’ but not ‘sativa’ (Figure S8). Within the cellular component category, of 19 significantly enriched GO terms in ‘sativa’ and ‘falcata’ genotypes, 10 were unique to ‘sativa’, while only 5 were significantly enriched in ‘falcata’ but not ‘sativa’ (Figure S9). 

### 2.7. Differential GO Term Enrichment between ‘Sativa’ and ‘Falcata’ under Drought Conditions

To further assess transcriptional differences between ‘sativa’ and ‘falcata’ genotypes, parametric analysis of gene set enrichment (PAGE), which takes into consideration both gene ID and log2 fold-changes, was carried out using DEGs derived from comparisons between ‘sativa’ and ‘falcata’ genotypes, under both well-watered and drought conditions. In the case of well-watered plants, no significant alterations in GO terms were observed between the two genotypes. 

**Figure 3:**
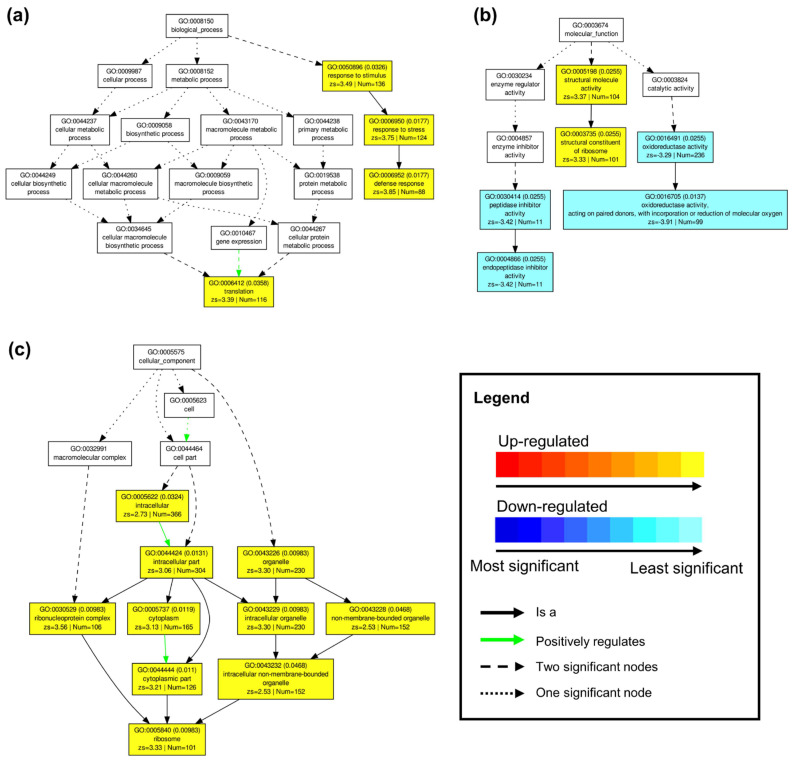
PAGE analysis of differentially expressed genes between drought-treated ‘sativa’ and ‘falcata’ genotypes. (**a**) Biological process GO term category. (**b**) Molecular function GO term category. (**c**) Cellular component GO term category. Analyses were carried out using AgriGO v2.0 using the Hochberg (FDR) multi-test adjustment method and a significance level of 0.05.

Conversely, substantial differences in GO term enrichment were noted between ‘sativa’ and ‘falcata’ genotypes under drought conditions (Figure 3). In the case of the biological process category, there was a significant enrichment of four up-regulated GO terms in ‘falcata’ compared to ‘sativa’ genotypes, including response to stimulus (GO:0050896), response to stress (GO:0006950), and defense response (GO:0006952) (Figure 3a). With respect to GO terms in the molecular function category, there was a significant enrichment of two up-regulated and four down-regulated GO terms in ‘falcata’ compared to ‘sativa’ (Figure 3b). Within the cellular component category, 10 significantly enriched GO terms were identified, all of which were up-regulated in ‘falcata’ compared to ‘sativa’ (Figure 3c).

### 2.8. Differential Transcriptional Responses in Abiotic Stress Response-Related Pathways between ‘Sativa’ and ‘Falcata’ in Response to Drought 

In order to gain a further understanding of the differential response of ‘sativa’ and ‘falcata’ genotypes to drought stress, we also carried out MapMan pathway analysis of DEGs between control and drought conditions in both ‘sativa’ and ‘falcata’, as well as between ‘sativa’ and ‘falcata’ plants under both control and drought conditions. 

**Figure 4:**
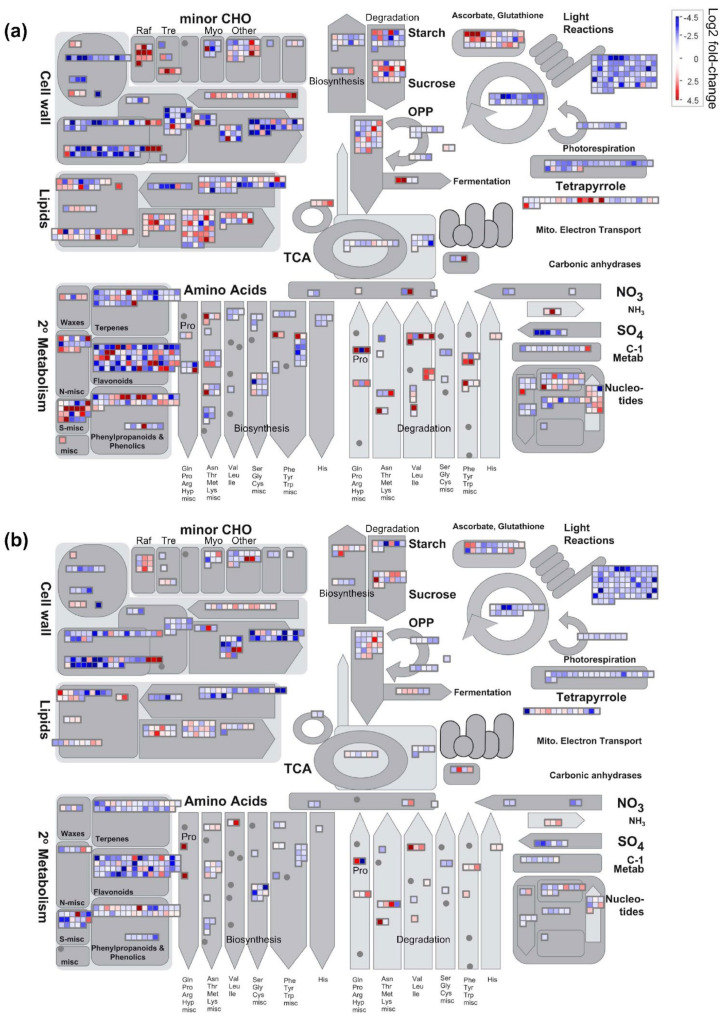
Transcriptional alterations in general metabolic pathways in ‘sativa’ (**a**) and ‘falcata’ (**b**) plants under control vs. drought conditions. Pathway analysis was conducted using MapMan, with blue boxes indicating down-regulated genes and red boxes denoting up-regulated genes. Myo, myo-inositol metabolism; Pro, proline metabolism; Raf, raffinose metabolism; Tre, trehalose metabolism.

**Figure 5:**
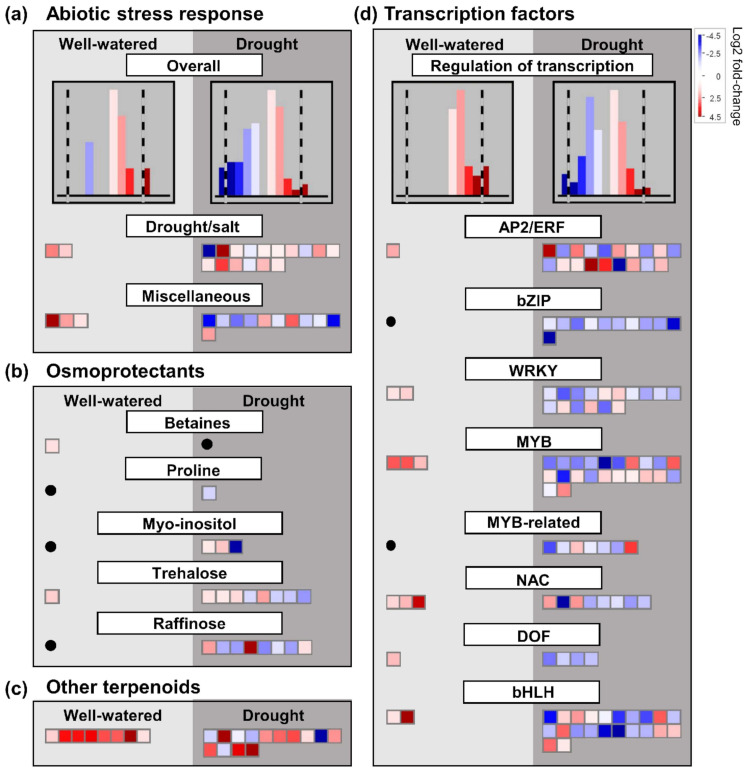
Transcriptional alterations in abiotic stress-related metabolic pathways and transcription factor families between ‘sativa’ and ‘falcata’ under both control and drought conditions. Pathway analysis was conducted using MapMan, with blue boxes indicating down-regulated genes and red boxes denoting up-regulated genes. Pathways shown include those related to (**a**) abiotic stress response, (**b**) the metabolism of various osmoprotectants, (**c**) the metabolism of non-tocopherol and non-carotenoid terpenoids, and (**d**) a selection of transcription factor families.

Between control and drought conditions, more DEGs were observed in ‘sativa’ than ‘falcata’ in most categories (Figure 4, Figures S10 and S11), including the ‘drought/salt’ and ‘miscellaneous abiotic stress’ categories (Figure S10, File S2). Between ‘sativa’ and ‘falcata’, a greater number of DEGs were observed under drought than control conditions in the vast majority of selected pathways with putative functions in abiotic stress response (Figure 5 and Figure S12). Under drought stress, 12 of 16 DEGs were up-regulated in ‘falcata’ compared to ‘sativa’ in the ‘drought/salt stress’ category (Figure 5a, File S2). Furthermore, there was a striking abundance of genes that were up-regulated in ‘falcata’ compared to ‘sativa’ overall under well-watered conditions, with all DEGs in the ‘drought/salt’ and ‘miscellaneous abiotic stress’ categories being up-regulated in ‘falcata’ compared to ‘sativa’ in this treatment (Figure 5a, File S2). Categories of a selection of differentially expressed stress response-associated genes are described below.

#### 2.8.1. Osmoprotectants

To decipher any putative role of osmoprotectants in the drought tolerance of ‘falcata’, we examined the expression of genes involved in their metabolism. Since proline accumulation does not appear to drive the enhancement in drought tolerance observed in ‘falcata’ compared to ‘sativa’ in the current study, we focused on the differential expression of genes involved in the metabolism of glycine betaine and minor carbohydrates such as myo-inositol, raffinose, and trehalose between treatments and genotypes. In ‘sativa’, a single betaine aldehyde dehydrogenase (Medtr3g078550) was up-regulated in response to drought, while no genes within this pathway exhibited differential expression between conditions in ‘falcata’ (Figure S10c,d, File S2). However, this same gene was significantly up-regulated in ‘falcata’ compared to ‘sativa’ under well-watered conditions (1.26081 log2 fold-change; Figure 5b, File S2). Taken together, this indicates that although ‘falcata’ did not significantly up-regulate the expression of this gene in response to drought, it was expressed at higher baseline levels compared to ‘sativa’.

With regard to genes involved in the metabolism of minor carbohydrates, ‘sativa’ exhibited a higher number of DEGs than ‘falcata’ between drought and control conditions in most categories (Figure 4, File S2). Of these, genes involved in myo-inositol metabolism were largely down-regulated, and those involved in raffinose metabolism were mostly up-regulated, under drought conditions in both genotypes. Conversely, while genes involved in trehalose metabolism were largely up-regulated under drought conditions in ‘sativa’, DEGs in this category were fairly evenly distributed between up- and down-regulated genes in ‘falcata’ (Figure 4; File S2). However, comparisons of DEGs between the two genotypes indicated that a single putative trehalose phosphatase/synthase (Medtr4g129270) was significantly up-regulated in ‘falcata’ compared to ‘sativa’ under well-watered conditions (Figure 5b, File S2). Numerous other genes involved in trehalose, myo-inositol, and raffinose metabolism were also found to be differentially expressed between ‘falcata’ and ‘sativa’ under drought conditions (Figure 5b, File S2). Of particular note, *GolS1* (Medtr1g084670), which has been previously implicated in raffinose series oligosaccharide (RSO) biosynthesis [16], was found to be up-regulated (2.22634 log2 fold-change) in ‘falcata’ compared to ‘sativa’ under drought conditions.

#### 2.8.2. Redox-Related Pathways

Due to the importance of scavenging and detoxifying ROS in plants under abiotic stress, we also surveyed DEGs with putative roles in this context. With respect to enzymatic antioxidants, a higher number of DEGs were observed overall in ‘sativa’ than ‘falcata’ in response to drought (Figures S10a,b and S12a, File S2). With respect to molecular antioxidants, the majority of DEGs encoding enzymes involved in tocopherol metabolism, and all DEGs involved in carotenoid metabolism, were down-regulated under drought compared to well-watered conditions in both genotypes (Figure S10c,d, File S2). In the case of other terpenoids, the majority were down-regulated under drought compared to well-watered conditions in both genotypes (Figure S10c,d, File S2). However, eight DEGs (Medtr5g024880, Medtr2g064425, Medtr2g089120, Medtr3g052120, Medtr4g045810, Medtr4g081460, Medtr6g039440, and Medtr6g093180) involved in terpenoid metabolism or the mevalonate pathway were expressed at significantly higher levels in ‘falcata’ compared to ‘sativa’ under control conditions, with no down-regulated genes observed. Four of these genes (Medtr5g024880, Medtr3g052120, Medtr4g045810, and Medtr4g081460) were also expressed at significantly higher levels in ‘falcata’ compared to ‘sativa’ under drought conditions (Figure 5c, File S2). Many genes involved in flavonoid metabolism were also differentially expressed between treatments in both genotypes (Figure 4), as well as between genotypes under both conditions (Figure S12a). The majority of these were expressed at lower levels in ‘falcata’ compared to ‘sativa’ in both growth conditions (File S2). 

#### 2.8.3. Hormone Metabolism

Between control and drought conditions, the ‘sativa’ genotype yielded a higher number of DEGs in the ABA, ethylene, brassinosteroid, and IAA metabolism categories than ‘falcata’, while ‘falcata’ displayed a greater number of DEGs in the salicylic acid, jasmonic acid, and gibberellin metabolism categories (Figure S10a,b, File S2). A 9-*cis*-epoxycarotenoid dioxygenase-encoding gene (Medtr2g070460), which typically functions as a rate-limiting enzyme in ABA and strigolactone biosynthesis (Ellison 2016), was up-regulated to a similar degree in both ‘sativa’ (log2 fold-change of 6.33952) and ‘falcata’ (log2 fold-change of 6.77675) under drought compared to well-watered conditions (File S2). In contrast, while a gene encoding an adenine nucleotide alpha hydrolase-like superfamily protein (Medtr1g054765), which was previously termed *MfUSP1* in *M. sativa* subsp. *falcata* [19] and falls within the ethylene-induced-regulated-responsive-activated bin in MapMan, was only up-regulated under drought conditions in ‘sativa’ (log2 fold-change of 1.57089; File S2), this gene was significantly up-regulated in ‘falcata’ compared to ‘sativa’ under both well-watered (3.43631 log2 fold-change) and drought (2.56688 log2 fold-change) conditions (Figure S12b, File S2).

#### 2.8.4. Transcription Factors

As was the case for most categories, increased numbers of transcription-factor-encoding DEGs were observed between control and drought conditions in ‘sativa’ than ‘falcata’ (Figure S11, File S2). Similarly, higher numbers of DEGs were observed between ‘sativa’ and ‘falcata’ under drought stress compared to well-watered conditions for all transcription factor categories assessed in this study (AP2/ERF, bZIP, WRKY, MYB, MYB-related, NAC, DOF, and bHLH) (Figure 5d, File S2). With the exception of AP2/ERF transcription factors, there was a predominance of genes that were down-regulated in ‘falcata’ compared to ‘sativa’ under drought conditions. Conversely, under well-watered conditions, all DEGs in these transcription factor categories were up-regulated in ‘falcata’ compared to ‘sativa’ (Figure 5d). For example, while *NAC3* (Medtr8g059170) was significantly up-regulated in both ‘sativa’ (log2 fold-change of 8.2973) and ‘falcata’ (log2 fold-change of 3.3731) under drought compared to well-watered conditions, this gene was significantly up-regulated in ‘falcata’ compared to ‘sativa’ under control conditions, while no differential expression was noted between the two genotypes under drought stress (File S2).

#### 2.8.5. Protective Proteins

In the current study, we found that a large number of genes belonging to three major classes of proteins with protective functions during drought response (heat shock proteins, LEAs (including dehydrins), and aquaporins) were differentially expressed in both ‘sativa’ and ‘falcata’ under drought compared to well-watered conditions (Figure S13). In the case of heat shock proteins, ‘sativa’ exhibited a higher number of DEGs than ‘falcata’ under drought compared to well-watered conditions, and an elevated number of DEGs were also noted between genotypes under drought than control conditions (Figure S13a). 

In the case of dehydrins, which are one class of LEA protein, three genes (Medtr3g117290, Medtr6g084640, and Medtr7g086340) were found to be significantly up-regulated under drought stress in both ‘sativa’ and ‘falcata’ (Figure S13b). Interestingly, one of these genes (Medtr6g084640) was significantly up-regulated in ‘falcata’ compared to ‘sativa’ under both growth treatments. In the case of other LEAs, most DEGs were up-regulated in both genotypes under drought compared to control conditions. Furthermore, a gene encoding LEA3 (Medtr4g123950), which was previously reported to be responsive to dehydration in *M. sativa* subsp. *falcata* [20], was found to be significantly up-regulated (2.596 log2 fold-change) in ‘falcata’ compared to ‘sativa’ under control conditions (Figure S13b). 

With respect to aquaporin-encoding genes, the vast majority were down-regulated in both genotypes under drought compared to control conditions (Figure S13c). While no differential expression was observed in these genes between genotypes under control conditions, several were differentially expressed under drought (Figure S13c).

## 3. Discussion

Previously, several *falcata* accessions were shown to exhibit superior drought tolerance or water use efficiency compared to the *sativa* subspecies; a phenomenon that has been suggested to be related to characteristics such as differences in root morphology, reduced stomatal density and conductance, delayed leaf senescence under drought, and increased RSO and (iso)flavonoid accumulation [22,24,25,26]. However, relatively few *falcata* accessions have been examined in-depth in terms of drought tolerance, and only a single *falcata* accession (Wisfal) has been comparatively assessed at the transcriptional level in this context thus far [24]. As such, we sought to compare various physiological, biochemical, and transcriptional characteristics in ‘sativa’ (Beaver) and ‘falcata’ (PI 641381) genotypes as a means of furthering our knowledge regarding the mechanisms driving improved resilience to water deficit in alfalfa.

In the current study, ‘falcata’ was found to exhibit superior drought tolerance compared to ‘sativa’, as evidenced by a significant reduction in the soil moisture level at which plants began to wilt (Figure 1a–b), and enhanced survival following severe drought (Figure 1f–g). In addition, while both ‘sativa’ and ‘falcata’ exhibited decreases in growth following drought recovery, which is a typical response of alfalfa [29,30], reductions were far more substantial in ‘sativa’ (Figure 2f–h). Given the mounting limitations in water resources, minimizing yield loss under drought conditions will be a crucial component of attaining both agricultural and environmental sustainability in alfalfa production [31,32]. 

Drought-induced senescence is commonplace among drought-sensitive plants and is typically correlated with a reduction in leaf chlorophyll content [24], which can lead to a decrease in photosynthetic capacity and eventually the death of the plant. Indeed, the ability to retain chlorophyll, and thus photosynthesis, under water deficit has been suggested to be a key contributor to drought tolerance in *falcata* genotypes assessed previously [24,25]. In the present study, we did not note any changes in leaf chlorophyll content between drought and well-watered conditions in either genotype (Figure S1b), and light-saturated photosynthetic rates declined under water deficit to a similar extent in both genotypes (Figure S1h), which was consistent with the down-regulation of a multitude of genes involved in the photosynthetic process (Figure 4). This implies that unlike previously studied *falcata* genotypes, chlorophyll content was not correlated with photosynthetic rate in this study, and neither trait appeared to contribute to the improvement of drought tolerance in ‘falcata’. 

The fact that ‘falcata’ plants wilted at a lower volumetric soil moisture content than ‘sativa’ and leaves retained a higher level of turgidity under severe drought stress (Figure 1) suggests that ‘falcata’ is better able to maintain a high water status under stress, thus avoiding dehydration and allowing for the continuation of metabolic function for a longer period of time. This corresponds with our finding that ‘sativa’ leaves exhibited a greater number of DEGs under drought compared to well-watered conditions than ‘falcata’ (Figure S4a), which resembles the outcome of a previous comparative microarray assessment of drought-tolerant Wisfal *falcata* and drought-sensitive Chilean *sativa* shoots [24]. However, PAGE analysis of our RNA-Seq data also indicated that up-regulated genes within the ‘response to stress’ category were significantly enriched in ‘falcata’ compared to ‘sativa’ under drought (Figure 3a). This signifies that while ‘sativa’ may have undergone an overall greater transcriptional response under drought than ‘falcata’, the latter exhibited the preferential regulation of genes known to be specifically involved in stress response. 

One way in which plants can avoid cellular dehydration under drought stress is through the superior capture of soil moisture [33], at least under certain water deficit scenarios. Accordingly, several *falcata* genotypes have been shown previously to exhibit higher root-to-shoot ratios under drought conditions than their *sativa* counterparts [24,25]. In the current study, root DW was lower in ‘falcata’ than ‘sativa’ under well-watered conditions. However, only ‘sativa’ exhibited a significant reduction in root length and DW following drought recovery (Figure 2k–m), which corresponds with the greater overall growth penalties incurred in this genotype. Therefore, although we did not examine root morphology in fine detail in this study, root size did not appear to be a main component of the improved drought tolerance in ‘falcata’. 

Another manner in which plant water status can be maintained under water-limited conditions is through a reduction in water loss. Given that ‘falcata’ took approximately 2 days longer to reach a soil moisture level of 1.5% (from approximately 50% soil moisture) than ‘sativa’ following drought treatment (Figure 1e), it is possible that this characteristic played a greater role in maintaining cellular hydration than enhanced soil moisture capture through the roots. Reductions in stomatal density and conductance are traits that have been associated with reduced water loss and are often linked to increased drought tolerance in many plant species, including alfalfa [24,25,30]. In the present study, we observed an increase in stomatal density in ‘falcata’ compared to ‘sativa’, but a decrease in stomatal width and area (Figure S1c–g), which corresponds with the lack of difference in stomatal conductance and transpiration rate on a per area basis between genotypes (Figure S1i–j). This is in stark contrast to a selection of *falcata* genotypes assessed previously, whereby they were found to exhibit decreases in stomatal density and/or stomatal conductance compared to *sativa* genotypes [24,25,26]. Although ABA levels, which can contribute to drought resilience in part through stomatal-related changes [12], were not examined in the current study, few differences were noted between genotypes with respect to the expression of ABA biosynthetic genes (File S2). Taken together, this implies that ABA-independent pathways and non-stomatal traits may be more important than ABA-dependent pathways in terms of eliciting superior drought tolerance in ‘falcata’.

Water loss can also be curtailed through non-stomatal leaf characteristics, such as a small leaf size and low SLA, and these traits are thus also associated with superior drought tolerance in plants [30,34,35]. In line with this, ‘falcata’ plants bore leaves with a significantly lower area and SLA than ‘sativa’ under well-watered conditions (Figure 2c–e). While we did not observe any difference between genotypes in detached leaf water loss assays (Figure S1a), which suggests that ‘falcata’ leaves were not inherently better at minimizing water loss than ‘sativa’ on a per weight basis, it is possible that the leaf characteristics of ‘falcata’ may be contributing to an overall improvement in transpiration efficiency on a whole-plant level. Such a phenomenon could also feasibly have been a factor in the slower rate of soil moisture utilization observed in these plants. 

An increase in the production of compatible solutes, such as proline, glycine betaine, and certain soluble sugars including trehalose and raffinose, within a plant under drought conditions can also have a positive impact on cellular hydration by augmenting osmotic pressure in the cytoplasm of plant cells, thus protecting enzymes and cell membranes under drought stress [36,37]. Proline accumulation did not appear to be a differentiating factor in drought resilience between ‘falcata’ and ‘sativa’ (Figure 4, Figure 5b and Figure S2a), which is consistent with previous findings in Wisfal *falcata* and Chilean *sativa* [24]. However, single genes involved in glycine betaine and trehalose biosynthesis, respectively, were up-regulated in ‘falcata’ compared to ‘sativa’ under well-watered conditions (Figure 5b, File S2). Similarly, *GolS1*, which encodes a galactinol synthase that catalyzes the production of galactinol that is utilized for the subsequent biosynthesis of RSO and elicits enhanced tolerance to drought, salinity, and cold tolerance when over-expressed in tobacco [16], was also up-regulated in ‘falcata’ compared to ‘sativa’ under drought conditions (File S2). Wisfal *falcata* was found previously to possess increased levels of several minor carbohydrates with known functions as osmoprotectants, including raffinose and myo-inositol, compared to Chilean *sativa* under both well-watered and water-limited conditions [24]. Together, these findings hint at the possibility that the accumulation of particular carbohydrates may be a general drought response mechanism across multiple *falcata* genotypes. Although total soluble carbohydrate levels were found to increase similarly under drought conditions in both ‘sativa’ and ‘falcata’ in the current study (Figure S2b), these changes do not necessarily reflect alterations in specific types of soluble carbohydrate, and it is thus feasible that an increase in the levels of these, and potentially other osmoprotectants may be a contributing factor to the enhanced ability of ‘falcata’ to maintain cell turgor under drought conditions.

In addition to a reduction in cellular hydration, drought stress also typically leads to an increase in the production of ROS such as superoxide (O_2_^-^) and hydrogen peroxide (H_2_O_2_). These ROS have important signaling functions in drought response, particularly through their function as secondary messengers that contribute to the coordination of specific physiological, molecular, and metabolic events [38]. However, above a certain threshold they have detrimental effects on cells and their components [36], and as such, plants typically increase the transcriptional levels/enzymatic activities of various enzymatic and non-enzymatic antioxidant systems under stress as a means of scavenging and detoxifying ROS [30,39]. The enzymatic antioxidant activity of SOD, APX, and CAT did not appear to contribute to the increased drought resilience of ‘falcata’ in this study (Figure S2c–e), and instead, both SOD and CAT activity increased to a greater extent in ‘sativa’ than ‘falcata’ under drought treatment. This implies that in line with the fact that ‘falcata’ was able to maintain a higher RWC than ‘sativa’ under the same intensity of water deficit, ROS levels may have been less impacted in the former genotype and there was thus less of a need to increase enzymatic antioxidant activity under these conditions. Interestingly, the transcriptional changes in genes encoding enzymatic antioxidants did not appear to follow this same pattern overall (Figures S10a,b and S12a, File S2). This was not wholly unexpected since the expression patterns of genes encoding antioxidant enzymes have not always been found to be congruent with fluctuations in enzyme activity under drought stress [40,41,42], which may be related to post-translational regulatory mechanisms that have yet to be fully elucidated [43,44]. 

Non-enzymatic antioxidants, such as flavonoids (Baskar et al. 2018) and isoprenoids including carotenoids [45], tocopherols [46], and a variety of other terpenoids [47], also function to reduce ROS levels during abiotic stress. In line with this, shoot flavonoids were found previously to be elevated in Wisfal *falcata* compared to Chilean *sativa* under both control and water-deficient conditions, with a concomitant increase in the propensity for the up-regulation of genes involved in their biosynthesis in the *falcata* genotype under drought compared to well-watered conditions [24]. However, a similar trend was not observed in the present study (Figure 4, File S2), and instead, a relatively large proportion of genes involved in the flavonoid biosynthetic pathway were expressed at lower levels in ‘falcata’ than ‘sativa’ under drought stress (Figure S12a). In agreement with our findings, a drought-tolerant alfalfa genotype was shown previously to exhibit a reduction in flavonoid levels under drought stress, whereas levels were not altered significantly in a drought-sensitive cultivar [32]. As such, it is possible that the role of these metabolites in the abiotic stress response may differ between species/genotypes [48,49], and that different alfalfa genotypes may employ distinct mechanisms to withstand drought stress. 

In the case of terpenoids, no obvious distinction was noted in the differential expression of genes involved in the metabolism of carotenoids or tocopherols between ‘sativa’ and ‘falcata’ (File S2). In contrast, we noted the significant up-regulation of eight genes involved in the metabolism of other terpenoids in ‘falcata’ compared to ‘sativa’ under well-watered conditions, while no down-regulated genes in this category were present (Figure 5c). Higher levels of metabolites involved in terpenoid biosynthesis were found in a drought-tolerant alfalfa genotype relative to a sensitive genotype under well-watered conditions previously [32], which is analogous to what we observed in the current study. As there has been a relative paucity of research attempting to unravel the role of non-carotenoid and non-tocopherol terpenoids in plants, it is possible that these specialized metabolites may provide an as of yet undeciphered mechanism contributing to drought tolerance in alfalfa. Therefore, while carotenoids and tocopherols do not likely contribute to the differential drought response observed between genotypes in the present study, further examination will be necessary to definitively determine whether baseline terpenoid levels/composition are involved. 

Differential levels of heat shock proteins, LEAs, and aquaporins have also been found to contribute to drought tolerance in plants [50]. Aquaporins function to regulate the movement of water across cellular membranes [51], and at least certain members of this family tend to be down-regulated in response to drought in plants, which may reduce water loss during dehydration [52]. In contrast, heat shock proteins and LEAs (including dehydrins), which function, at least in part, as molecular chaperones to maintain protein stability and cellular homeostasis, are both typically up-regulated in response to drought stress [53,54]. Genes encoding all three types of protein have been shown to follow this trend at the transcriptional level in Wisfal *falcata* and Chilean *sativa*, with many being more responsive to drought in *sativa* than *falcata* [24]. While we observed a similar pattern in the current study, there were several exceptions (Figure S13). Of particular note was a gene encoding LEA3, which was significantly up-regulated in ‘falcata’ compared to ‘sativa’ under control, but not drought, conditions (Figure S13b). Previously, the over-expression of *MfLEA3* (derived from a *falcata* accession) in tobacco enhanced tolerance to drought, cold, and high light, possibly due in part to a concomitant decrease in ROS accumulation [20]. Therefore, it is possible that higher levels of baseline expression of this gene may play a role in the superior drought tolerance observed in ‘falcata’ compared to ‘sativa’ via a protective effect on as of yet unidentified proteins.

Alterations in the expression of genes encoding particular transcription factors have also been shown to be important for the regulation of drought stress response [55]. In the present study, we found that overall, genes encoding transcription factors were more highly regulated in ‘sativa’ than ‘falcata’ under drought compared to control conditions (Figure S11). Intriguingly, we also found that despite the greater overall response of ‘sativa’ under drought in this context, all differentially expressed genes encoding abiotic stress-related transcription factors assessed in this study were up-regulated in ‘falcata’ compared to ‘sativa’ under well-watered conditions (Figure 5d), which implies that ‘falcata’ might possess higher baseline levels of these transcription factors than ‘sativa’. It is noteworthy that, in certain cases, drought tolerance can be attributed to the enhanced expression of genes, including a subset of transcription factors, prior to the onset of drought, thus rendering the plant quicker to respond [56]. Accordingly, *NAC3*, which was shown previously to positively regulate cold tolerance in *M. truncatula* [57], was up-regulated in ‘falcata’ compared to ‘sativa’ under control but not drought conditions (File S2).

Finally, we also found that the expression of *USP1* was significantly higher in ‘falcata’ than ‘sativa’ under both well-watered and drought conditions (File S2). The heterologous expression of a *falcata* homolog of this gene in tobacco was previously shown to enhance tolerance to various types of abiotic stress, including osmotic stress, at least in part by lowering ROS accumulation [19]. Although the precise mechanism by which this gene elicits improvements in abiotic stress tolerance remains to be determined, it is possible that its increased expression may also influence drought response in ‘falcata’.

In conclusion, unlike the small number of *falcata* genotypes assessed previously [24,25,26], ‘falcata’ PI 641381 did not appear to elicit its superior drought resilience through alterations in stomatal-related traits, root size, or delayed senescence under drought. While ‘falcata’ may make use of increased RSO accumulation, as evidenced by the up-regulation of *GolS1* in ‘falcata’ compared to ‘sativa’ under drought conditions, which resembles one possible factor behind improved drought tolerance in the Wisfal *falcata* genotype [24], it also appears to utilize a unique suite of additional mechanisms to achieve drought resilience. These putative mechanisms include the presence of smaller, thicker leaves and an increase in baseline transcriptional levels of a number of genes under well-watered conditions, which could feasibly allow a more rapid response to drought. This suggests that different *falcata* accessions/genotypes may make use of distinct mechanisms to enhance their ability to thrive under drought conditions. While the majority of studies to date have focused on common drought response mechanisms in particular alfalfa accessions/cultivars, little effort has been directed towards elucidating differences between drought-tolerant genotypes thus far. However, such discrepancies have been found to occur in other plant species [58,59], highlighting the complexity of drought tolerance mechanisms in general. As such, deciphering the molecular and regulatory processes driving the superior ability of *falcata* genotypes to withstand adverse conditions will provide knowledge and molecular tools to improve alfalfa, and potentially also other crops, in the future.

## 4. Materials and Methods

### 4.1. Plant Growth Conditions

Seeds of tetraploid *M. sativa* subsp. *falcata* accession PI 641381 (‘falcata’; determined to exhibit superior resilience to drought stress in preliminary experiments) were obtained from the United States Department of Agriculture—Agricultural Research Service Germplasm Resources Information Network (https://www.ars-grin.gov/Pages/Collections; accessed on 16 October 2017). This accession was derived from a population grown in Russia at a latitude of 56°. Seeds of *M. sativa* subsp. *sativa* cv. Beaver (‘sativa’), which is relatively susceptible to drought and is typically grown under irrigation [60], were provided by Dr. Surya Acharya (Agriculture and Agri-Food Canada, Lethbridge Research and Development Centre, Lethbridge, Canada). Due to the outcrossing nature of *M. sativa*, all assessments were carried out using biological replicates (details are provided in specific subsections below, as well as in figure legends) of a single genotype of each accession/cultivar derived from vegetative stem cuttings for each treatment, respectively. 

All plants were grown in Cornell mix in 4” pots under greenhouse conditions with supplemental light with a 16 h/8 h photoperiod, and a day/night temperature of approximately 20/15 °C. Pots were rotated daily to prevent microclimate effects and plants were cut back to approximately 5 cm at least twice prior to assessments. Drought treatment involved withholding water and daily monitoring of volumetric soil moisture content using a ML3 ThetaKit soil moisture meter (Hoskin Scientific Ltd., Burnaby, Canada), as well as stress symptoms, such as dry shoots and wilted leaves. All pots were adjusted to a soil moisture content of 50% prior to the experiment. All physiological, biochemical, and transcriptomic analyses were carried out at volumetric soil moisture contents of approximately 50% (control treatment) and 7–8% (drought treatment; when ‘sativa’ plants first began exhibiting signs of dehydration stress). All growth measurements were carried out using well-watered plants (approximately 50% volumetric soil moisture content) and in certain cases also following drought recovery (2–3 weeks of re-watering after allowing volumetric soil moisture contents to reach approximately 4% (aboveground measurements) or 1% (root measurements)). 

### 4.2. Assessment of Growth Characteristics

Between ten and eleven vegetative stem cuttings for each treatment (drought recovery and control) were generated from a single genotype of ‘sativa’ and ‘falcata’, respectively, in order to assess growth characteristics and penalties in each case. Flowering time was defined as the number of days following cutting to the appearance of the first open flower in well-watered plants. 

To evaluate growth penalties following drought recovery, aboveground and belowground plant growth characteristics were assessed. For aboveground evaluations, measurements were carried out 35–37 days after cutting under well-watered control conditions (soil moisture levels were maintained at approximately 50%) and following drought recovery (soil moisture content was allowed to reach approximately 4%, after which time plants were re-watered for 2 weeks). Plant height was derived from the length of the longest shoot, internode length consisted of the mean value of the longest internode on the three longest shoots of each plant, and the number of shoots comprised the total number of primary, secondary, and tertiary shoots per plant. Aboveground FW was evaluated by weighing all aboveground tissue immediately following harvest, while DW was determined following drying at 65 °C for at least 1 week.

Root assessments were carried out 54 days after cutting under well-watered conditions (control) and following drought recovery, which comprised allowing soil moisture content to reach approximately 1%, followed by re-watering for 3 weeks. At the time of evaluation, plants were removed from their pots and the roots were washed thoroughly. Root length was determined from the longest root on each plant. Root DW measurements were determined by drying at 65 °C for at least 1 week prior to weighing. 

For evaluation of survival following drought treatment, water was withheld until volumetric soil water content reached approximately 1%, after which time plants were re-watered normally for 2–3 weeks and plant survival was assessed by determining the percentage of plants that regenerated.

### 4.3. Leaf Characteristics and Stomatal Measurements

Leaf length, leaf width, leaf area, and SLA were measured 21 days after cutting on five biological replicate plants of each genotype, with three leaves assessed per plant, using the middle leaflet of the third fully expanded trifoliate leaf from the shoot tip. Leaf area was resolved using the Petiole plant leaf area meter app (version 2.0.1; https://play.google.com/store/apps/details?id=com.petioleapp.petiole&hl=en_CA&gl=US; accessed on 29 April 2019) and leaf dry weight was then determined after drying at 80 °C for 24 h. Specific leaf area was established by dividing leaf area by dry weight in each case. 

Stomatal density, length, width, and area were measured using middle leaflets of third trifoliate leaves (from the shoot tip) from three biological replicate 35-day-old plants. Stomatal density was determined by applying clear nail polish to the abaxial side of leaflets (four to five leaflets from each of the three biological replicate plants), which were then allowed to dry for 10–15 min. The leaf imprints were then peeled off with the help of transparent tape, and slides were prepared. The resulting slides were visualized with an EVOS FL Auto Imaging System (Thermo Fisher Scientific, Waltham, MA, USA) under 20× magnification and stomata were counted. Values were then converted to the number of stomata per mm^2^ in each case. Stomatal length, width, and area were assessed on six to seven randomly selected stomata from each of the three biological replicate plants (twenty stomata total for each genotype) using the EVOS FL Auto Cell Imaging System software (Thermo Fisher Scientific, Waltham, MA, USA).

### 4.4. Measurement of Relative Water Content and Detached Leaf Water Loss

Water deficit was estimated by measuring the RWC of first fully expanded trifoliate leaves from ten biological replicates of each genotype, as described previously [61], when soil moisture content reached approximately 50% (well-watered), 20% (mild drought), and 7% (drought). In brief, the fresh weight (FW) of trifoliate leaves was recorded immediately after harvesting, turgid weights (TW) were resolved after submersing petioles in water in an enclosed Eppendorf tube for 3–4 h, and dry weights (DW) were established by drying turgid leaves at 80 °C overnight. RWC (%) was then calculated as [(FW − DW)/(TW − DW)] × 100. 

Detached leaf water loss assays were carried out on five biological replicate plants of ‘sativa’ and ‘falcata’ by weighing a fully expanded trifoliate leaf from each plant immediately upon harvest (W_initial_), placing the leaf in the open air on a benchtop, and then weighing every 30 min for 180 min. The rate of water loss as a percentage was calculated as (W_initial_ − W_at particular time_/W_initial_) × 100.

### 4.5. Biochemical Assessments

All biochemical assessments were conducted using first fully expanded trifoliate leaves from nine to ten biological replicates of each genotype in each treatment. In all instances, leaf tissue was freeze-dried prior to carrying out assays. The control treatment comprised plants with soil moisture levels maintained at approximately 50%, while drought-treated samples were harvested when soil moisture levels reached approximately 7% (when ‘sativa’ plants were beginning to show signs of drought stress). 

Total soluble sugar content was assessed using the Plant Soluble Sugar Content Assay Kit according to the manufacturer’s instructions (MyBioSource Inc., San Diego, CA, USA). Two technical replicates were carried out for each sample. Proline content was determined as described previously [62] with minor modifications. In brief, approximately 50 mg (FW) leaf tissue was freeze dried and ground using a TissueLyzer II (Qiagen Inc., Toronto, ON, Canada), after which time 1 mL 3% aqueous sulphosalicylic acid was added to each tube. The tissue was further homogenized and incubated at room temperature for 3 h, and then centrifuged at 1500× *g* for 10 min. Following centrifugation, 600 μL of sample supernatant (or various concentrations of L-proline standard) was added to 600 μL glacial acetic acid and 600 μL ninhydrin reagent (0.025 g/mL ninhydrin, 0.6 mL/mL glacial acetic acid, 2. 4M H_3_PO_4_). Reactions were incubated at 100 °C for 45 min, then cooled on ice for 30 min. To each tube, 1.2 mL toluene was added, and reactions were vortexed and then centrifuged at 1000× *g* for 5 min. Proline content was determined in triplicate in microplate format using toluene as a blank in a Synergy Mx Multi-Mode Microplate Reader spectrophotometer (BioTek Instruments Inc., Winooski, VT, USA) at 520 nm.

### 4.6. Evaluation of Antioxidant Activity

Antioxidant assays were carried out using a single first fully expanded trifoliate leaf from nine to ten biological replicates of ‘sativa’ and ‘falcata’ plants under well-watered (approximately 50% soil water content) and drought (approximately 7% soil water content) conditions. Tissue was immediately frozen in liquid nitrogen, ground in a TissueLyzer for 1 min at 30 Hz, and placed back in liquid nitrogen. To each sample, 1.5 mL extraction buffer (0.15 M potassium phosphate buffer, pH 7.8 and 1 mM EDTA) was added and samples were vortexed for 30 s. Subsequently, the samples were centrifuged at 12,000× *g* at 4 °C for 20 min, and the supernatant was transferred to a fresh tube. Extracts were used for all subsequent assays, which were carried out in triplicate. Protein concentration was determined using a Bradford assay [63], with 4 μL extract and the Quickstart Protein Assay according to the manufacturer’s microassay protocol, with bovine serum albumin as a standard (Bio-Rad Laboratories Ltd., Hercules, CA, USA).

CAT activity was evaluated as described previously [64] with minor modifications. Initially, the spectrophotometer (SmartSpec Plus; Bio-Rad Laboratories Ltd., Hercules, CA, USA) was blanked at 240 nm with 50 mM potassium phosphate buffer (pH 7.0). For each assay, 33 μL sample extract was added to 967 μL CAT reaction mixture (50 mM potassium phosphate buffer, pH 7.0, 10 mM H_2_0_2_) in a 1 mL cuvette and the absorbance was immediately measured at 240 nm (A_sampleT0_). A second reading was carried out after precisely 3 min (A_sampleT3_). CAT activity (nM H_2_0_2_ · min^−1^ · mg protein^−1^) was calculated as (A_sampleT0_ − A_sampleT3_)/(e × d × t × C), where e corresponds to 39.4 M^−1^ (extinction coefficient of H_2_0_2_), d corresponds to 1 cm (cuvette path length), t corresponds to 3 min (incubation time), and C corresponds to the amount of protein (mg) within the 33 μL of extract used for analysis.

APX activity was determined as described previously [65] with minor modifications. To a 1 mL cuvette, 967 μL APX reaction mixture (50 mM potassium phosphate buffer, pH 7.0, 0.5 mM ascorbic acid, 0.1 mM EDTA) was mixed with 33 μL sample extract and 5 μL 200 mM H_2_0_2_. APX reaction buffer was used in place of sample extract for blanks. Absorbances were read immediately at 290 nm (A_sampleT0_), and a second reading was taken after precisely 3 min (A_sampleT3_). APX activity (μM ascorbate · min^−1^ · mg protein^−1^) was calculated as (A_sampleT0_ − A_sampleT3_)/(e × d × t × C), where e corresponds to 2.8 mM^−1^ · cm^−1^ (extinction coefficient of H_2_0_2_), d corresponds to 1 cm (cuvette path length), t corresponds to 3 min (incubation time), and C corresponds to the amount of protein (mg) within the 33 μL of extract used for analysis.

SOD activity was assessed as described previously [66] with minor modifications to allow for microplate format. Briefly, each reaction consisted of 5 μL of sample extract and 195 μL SOD reaction mixture (50 mM potassium phosphate buffer, pH 7.8, 2 mM EDTA, 9.9 mM L-methionine, 55 μM nitroblue tetrazolium, 0.025% Triton X-100 and 1 μM riboflavin). Every reaction was replicated under light (approximately 80 μmol m^−2^ s^−1^) and dark conditions, and was incubated for 10 min at room temperature in each case. Absorbance was then measured with a microplate spectrophotometer (Synergy Mx Multi-Mode Microplate Reader; BioTek Instruments Inc., Winooski, VT, USA) at 560 nm with extraction buffer as a blank (A_blank_). Sample absorbance (A_sample_) was calculated as absorbance in the dark subtracted from absorbance in the light. SOD activity (SOD units/mg protein) was calculated as [(A_blank_ − A_sample_)/(A_blank_ × 0.5)]/mg protein.

### 4.7. Chlorophyll- and Photosynthesis-Related Measurements

Chlorophyll content in the leaves of ten to eleven biological replicates of ‘sativa’ and ‘falcata’ genotypes was determined using a CCM-200 Chlorophyll Content Meter (Hoskin Scientific Ltd., Burlington, ON, Canada). The middle leaflet of third fully expanded trifoliate leaves was used for measurements in each case, with the values obtained from three leaves averaged for each biological replicate. Leaves were assessed under well-watered conditions (approximately 50% soil moisture content) and when drought-treated ‘sativa’ plants were just beginning to display symptoms of stress (soil moisture content of approximately 8%). 

Stomatal conductance (*g*_s_), transpiration rate (E), and light-saturated photosynthetic rate (Asat) were measured with an LI-6800 (LI-COR Inc., Lincoln, NE, USA). The centre leaflet of a first fully expanded dark green trifoliate leaf was used for measurements, and all observations were carried out between 12:45 pm and 3:45 pm in the greenhouse. Leaves were evaluated under well-watered conditions (approximately 50% soil moisture content) and under drought treatment (average soil moisture content of approximately 7%). Four biological replicates were utilized for ‘sativa’ under drought conditions and eleven biological replicates were used for the remaining groups. The number of ‘sativa’ drought-treated plants assessed was lower than other groups due to the lack of viable tissue in a proportion of plants as a result of drought stress on the day testing was carried out. Within the chamber, light intensity was maintained at 1500 μmol m^−2^ s^−1^, relative humidity at 65%, air temperature at 22 °C, and CO_2_ level at 410 μmol CO_2_/mol air. Each leaflet was allowed 3 min to stabilize within the chamber prior to assessment. All three measurements were adjusted for leaf area, which was determined using the Petiole app (version 2.0.1) as described in a previous section. 

### 4.8. RNA-Seq Data Analysis

#### 4.8.1. Sequencing and Annotation

Leaf tissue was harvested from control (soil moisture content of approximately 50%) and drought-treated plants (soil moisture content of approximately 7%), flash frozen in liquid nitrogen, and stored at −80 °C. Tissue was harvested from four biological replicates of ‘sativa’ and ‘falcata’ under each treatment, respectively. Total RNA was extracted from ground tissue using the Spectrum Plant Total RNA Kit according to the manufacturer’s instructions (Sigma-Aldrich Corp., St. Louis, MO, USA). RNA integrity was confirmed by resolving a small aliquot on a 1% agarose gel and using a 2100 BioAnalyzer (Agilent Technologies, Santa Clara, CA, USA). A stranded mRNA library was prepared using 250 ng of total RNA and the NEBNext® system (New England Biolabs Ltd., Whitby, ON, Canada), and sequencing was carried out using an Illumina NovaSeq 6000 platform (Illumina Inc., San Diego, CA, USA) with 100 bp paired-end reads by a third party (Genome Québec Centre d’Expertise et de Services, Montreal, QC, Canada). The resulting raw RNA-Seq data was analyzed as described previously [67] with minor modifications. Briefly, raw reads were trimmed using the ‘sickle’ script in Linux with default parameters, and read quality was assessed using the FASTQC tool (https://www.bioinformatics.babraham.ac.uk/projects/fastqc/; accessed on 30 May 2020). High-quality filtered reads were mapped to the *Medicago truncatula* genome (Mt4.0 v2; http://www.medicagogenome.org/downloads; accessed on 31 May 2020; a close diploid relative of alfalfa with a well-annotated genome sequence) using Tophat2 [68]. 

DEGs were identified and normalized to FPKM using Cuffdiff [69]. Genes with a false discovery rate (FDR) [70] of less than 0.05 were considered DEGs (File S1). PCA was performed using total exon read counts, which were obtained using the featureCounts program [71], followed by analysis using freely available R-software (v4.0). Venn diagrams were generated using freely available software (http://bioinformatics.psb.ugent.be/webtools/Venn/; accessed on 15 June 2021). The sequence data generated in this study are available at the National Center for Biotechnology Information (NCBI) Sequence Read Archive (BioProject accession number PRJNA765383).

#### 4.8.2. GO Term Enrichment and Pathway Analysis

SEA of either up-regulated or down-regulated DEGs observed between control and drought conditions for both ‘sativa’ and ‘falcata’ were carried out using AgriGO v2.0 (http://systemsbiology.cau.edu.cn/agriGOv2/; accessed on 13 January 2021) [72] with *M. truncatula* as the reference species. In both cases, the hypergeometric statistical test was used, along with the Yekutieli (FDR under dependency) multi-test adjustment method, with a significance level of 0.05. SEACOMPARE was conducted by inputting SEA results. PAGE was carried out by inputting all up-regulated and down-regulated DEGs observed between ‘sativa’ and ‘falcata’ when grown either under control or drought conditions, along with log2 fold-change expression values (expression values of 0 were artificially set to 0.001 in order to provide numerical log2 fold-changes), using the same program with the Hochberg (FDR) multi-test adjustment method and a significance level of 0.05. Heat maps were generated using the freely available Morpheus tool (https://software.broadinstitute.org/morpheus/; accessed on 20 May 2021). Visualization of DEG-associated pathways between ‘sativa’ and ‘falcata’ under both well-watered and control conditions was performed using MapMan V3.6 software (https://mapman.gabipd.org/) with the *M. truncatula* genome as a reference sequence (Mt4.0 v2). 

### 4.9. Validation of RNA-Seq Results

Total RNA that was extracted from leaf tissues for RNA-Seq analysis was utilized for qRT-PCR validation. First-strand cDNA synthesis was carried out using the SuperScript VILO cDNA synthesis kit (Thermo Fisher Scientific, Waltham, MA, USA) and quantitative real-time RT-PCR assays were conducted using an appropriate dilution of each cDNA template along with PerfeCTa SYBR Green Supermix (VWR International LLC, Mississauga, ON, Canada) in a final reaction volume of 10 µL. Assays were accomplished on a Quantstudio 6 Flex Real-Time PCR System (Thermo Fisher Scientific, Waltham, MA, USA) using primers designed to anneal to a region of coding sequence for ten genes selected based on their up- or down-regulation in RNA-Seq analyses (see Table S2 for primer sequences used for qRT-PCR assays). A 183 nt region of the constitutively expressed actin-depolymerizing factor (*ADF*) gene, which has been shown previously to act as a highly stable reference gene for qRT-PCR across developmental stages and environmental conditions (including water stress) in alfalfa [73], was amplified as an internal control. Thermal parameters for amplification were as follows: initial denaturation at 95 °C for 3 min, followed by 40 cycles of 95 °C for 15 s and 60 °C for 45 s. Dissociation curves were generated to confirm the presence of a single amplification product in each case. Levels of gene expression were established using the standard curve method and Applied Biosystems^TM^ analysis software v4.0 (Thermo Fisher Scientific, Waltham, MA, USA), with the expression of each target gene comprising mean values of four biological replicates (three technical replicates each) normalized to that of the internal control. Log2 fold-change values between control and drought samples were calculated for comparison to RNA-Seq values.

### 4.10. Statistical Analyses

For the majority of multi-variate comparisons, the observed response variables were modeled using the GLIMMIX procedure in SAS (SAS version 9.4, SAS Institute Inc., Cary, NC, USA) with one-thousand iterations at multiple levels of iteration (MAXOPT = 1000 and NLOPTIONS MAXITER = 1000). The normal distribution of the response was not assumed and therefore the models were “generalized.” The best-fitting distribution from the exponential family of distributions (e.g., gamma, inverse Gaussian, lognormal, shifted-*t*, normal, and exponential) was selected for each variable, based on the model fit statistics, that is, the Bayesian information criterion (BIC). The models were “mixed” due to the inclusion of fixed factors (*Genotype, Growth_conditions,* and *Genotype* *× Growth_conditions*) and random factors (*Biological_replicate*). Variance homogeneity was not assumed and models of variance heterogeneity were tested and selected based on the BIC of the models. Bonferroni’s method was used to adjust for multiple comparisons. For assessments that involved comparisons between genotypes under a single treatment, as well as for evaluations of photosynthetic rate, stomatal conductance, and transpiration rate, 2-tailed Student’s *t*-tests assuming unequal variance were used for statistical analysis. Differences were considered significant at *p* ≤ 0.05.

## Data Availability

Sequence data generated in this study is available at the National Center for Biotechnology Information (NCBI) Sequence Read Archive (BioProject accession number PRJNA765383).

## Supplementary Materials

The following are available online at www.mdpi.com/article/10.3390/plants10102107/s1, Figure S1: Stomatal, photosynthetic, and water loss-related traits in ‘sativa’ and ‘falcata’ plants, Figure S2: Biochemical and antioxidant response of ‘sativa’ and ‘falcata’ genotypes to drought stress conditions, Figure S3: Principal component analysis of FPKM expression values, Figure S4: Identification of DEGs between genotypes and conditions and validation of RNA-Seq results, Figure S5: SEACOMPARE analysis of up-regulated DEGs observed in ‘sativa’ control vs. drought (1) and ‘falcata’ control vs. drought (2) in the biological process GO grouping, Figure S6: SEACOMPARE analysis of up-regulated DEGs observed in ‘sativa’ control vs. drought (1) and ‘falcata’ control vs. drought (2) in the molecular function GO grouping, Figure S7: SEACOMPARE analysis of down-regulated DEGs observed in ‘sativa’ control vs. drought (1) and ‘falcata’ control vs. drought (2) in the biological process GO grouping, Figure S8: SEACOMPARE analysis of down-regulated DEGs observed in ‘sativa’ control vs. drought (1) and ‘falcata’ control vs. drought (2) in the molecular function GO grouping, Figure S9: SEACOMPARE analysis of down-regulated DEGs observed in ‘sativa’ control vs. drought (1) and ‘falcata’ control vs. drought (2) in the cellular compartment GO grouping, Figure S10: Transcriptional alterations in the abiotic stress response, as well as phytohormone-, redox-, and secondary-metabolism-related pathways in ‘sativa’ (a and c) and ‘falcata’ (b and d) plants under control vs. drought conditions, Figure S11: Transcriptional alterations in genes involved in transcriptional regulation in ‘sativa’ (a) and ‘falcata’ (b) plants under control vs. drought conditions, Figure S12: Transcriptional alterations in redox-related pathways, as well as hormone metabolism, between ‘sativa’ vs. ‘falcata’ under control and drought conditions, Figure S13: Differential expression of genes encoding heat shock factors, LEAs, and aquaporins between conditions and genotypes, Table S1: RNA-Seq read and alignment data for ‘sativa’ and ‘falcata’ under well-watered (control) and drought conditions, Table S2: Primers used for qRT-PCR validation of RNA-Seq results, File S1: Differentially expressed genes between genotypes and growth conditions, File S2: Information regarding differentially expressed genes between treatments and genotypes falling into a selection of MapMan categories with putative functions in abiotic stress response.
