# Supplementary material for: Identification of Differential Drought Response Mechanisms in Medicago sativa subsp. sativa and falcata through Comparative Assessments at the Physiological, Biochemical, and Transcriptional Levels
Source: Plants (Basel). 2021 Oct 5;10(10):2107. doi: 10.3390/plants10102107 (PMC8539336; doi:10.3390/plants10102107)
Supplement: Supplementary file 1 [file plants-10-02107-s001.zip › Supplemental Figure 1 Stomatal and photosynthetic parameters (Aug 23 2021).pdf]

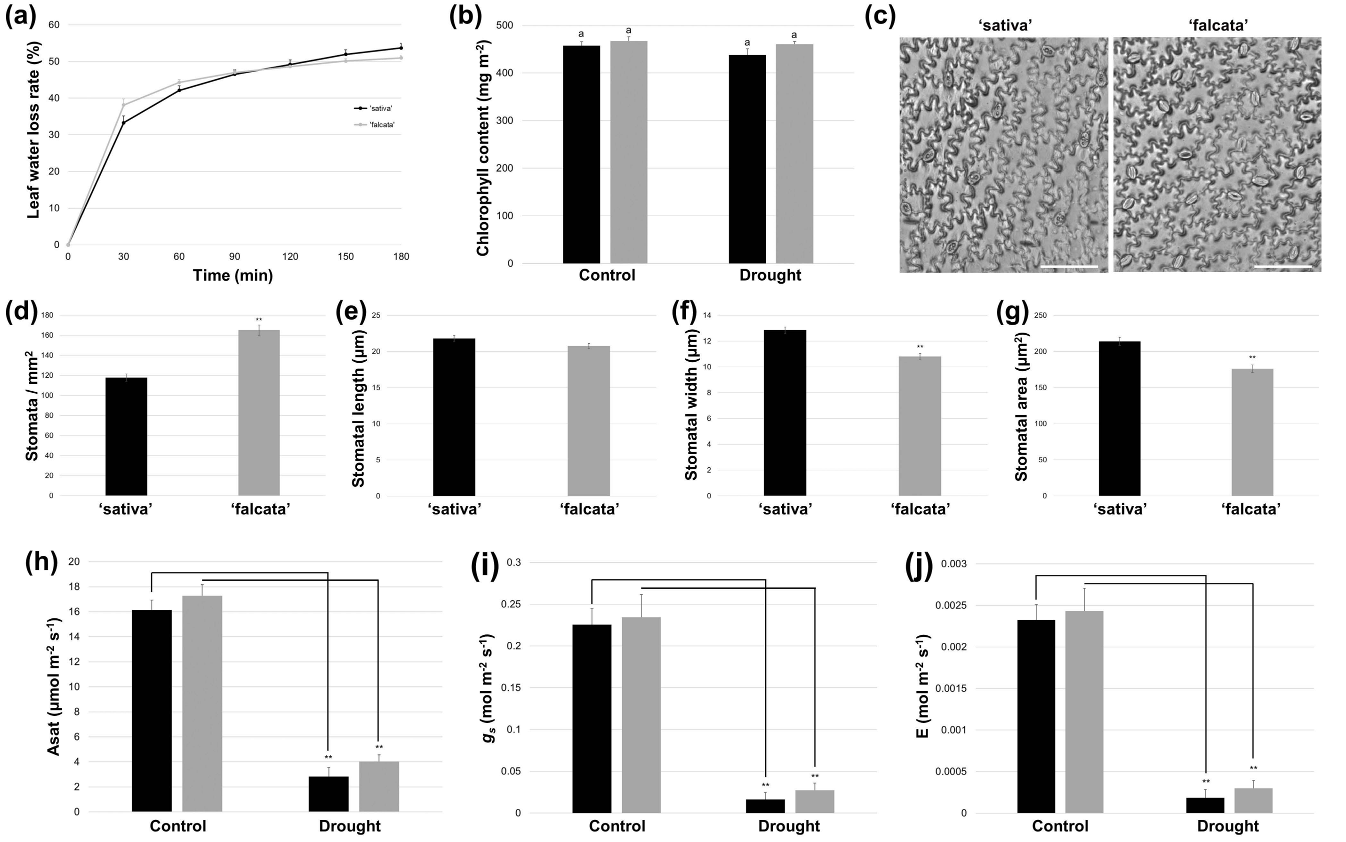

Figure S1. Stomatal, photosynthetic and water loss-related traits in 'sativa' and 'falcata' plants. In all graphs, 'sativa' is represented in black while 'falcata' is represented in gray. **(a)** Rate of water loss from detached leaves of 'sativa' and 'falcata' genotypes. Fully expanded trifoliate leaves were harvested and subjected to water loss rate assays over 180 minutes. Each data point represents the mean value of 5 biological replicates derived from vegetative stem cuttings. Bars denote standard errors. Student's *t*-tests (two-sided, unequal variance) were carried out for each time point; however, no significant differences ($p \leq 0.05$) were observed. **(b)** Chlorophyll content in the middle leaflet of third trifoliate leaves from the shoot tip under well-watered (soil moisture content of approximately 50%) and drought (soil moisture content of approximately 8%) conditions. Blocks consist of the mean value of 10-11 biological replicates derived from vegetative stem cuttings. **(c)** Representative images of the abaxial side of the middle leaflet of the third fully expanded trifoliate leaf from 'sativa' and 'falcata' genotypes. Scale bar = 100 µm. **(d-g)** Stomatal density, length, width and area were assessed on middle leaflets of third trifoliate leaves from 'sativa' and 'falcata' plants. Each block represents the mean of 30 ('sativa') and 38 ('falcata') biological replicates for stomatal density, and 20 randomly selected stomata for length, width and area. **(h-j)** Light saturated photosynthetic rate (Asat), stomatal conductance (g_s) and transpiration rate (E) were assessed using the middle leaflet of a first fully expanded trifoliate leaf under well-watered (soil moisture content of approximately 50%) and drought (average soil moisture content of approximately 7%) conditions. All measurements were adjusted for leaf area. Blocks in each graph represent the mean value of 4-11 biological replicates derived from stem cuttings. For all graphs, bars denote standard errors with black blocks representing 'sativa' and gray representing 'falcata'. Lowercase letters indicate statistically significant differences among groups in each graph ($p \leq 0.05$) as determined through the generation of mixed generalized models using the GLIMMIX procedure in SAS, and the subsequent use of Bonferroni's method to adjust for multiple comparisons. Asterisks denote means that are significantly different from the indicated group as determined by 2-tailed Student's *t*-tests assuming unequal variance (**, $p \leq 0.01$).
