# Supplementary material for: Identification of Differential Drought Response Mechanisms in Medicago sativa subsp. sativa and falcata through Comparative Assessments at the Physiological, Biochemical, and Transcriptional Levels
Source: Plants (Basel). 2021 Oct 5;10(10):2107. doi: 10.3390/plants10102107 (PMC8539336; doi:10.3390/plants10102107)
Supplement: Supplementary file 1 [file plants-10-02107-s001.zip › Supplemental Figure 10 Abiotic Stress Phytohormones Redox Secondary Mapman control vs drought sativa and falcata (May 21 2021).pdf]

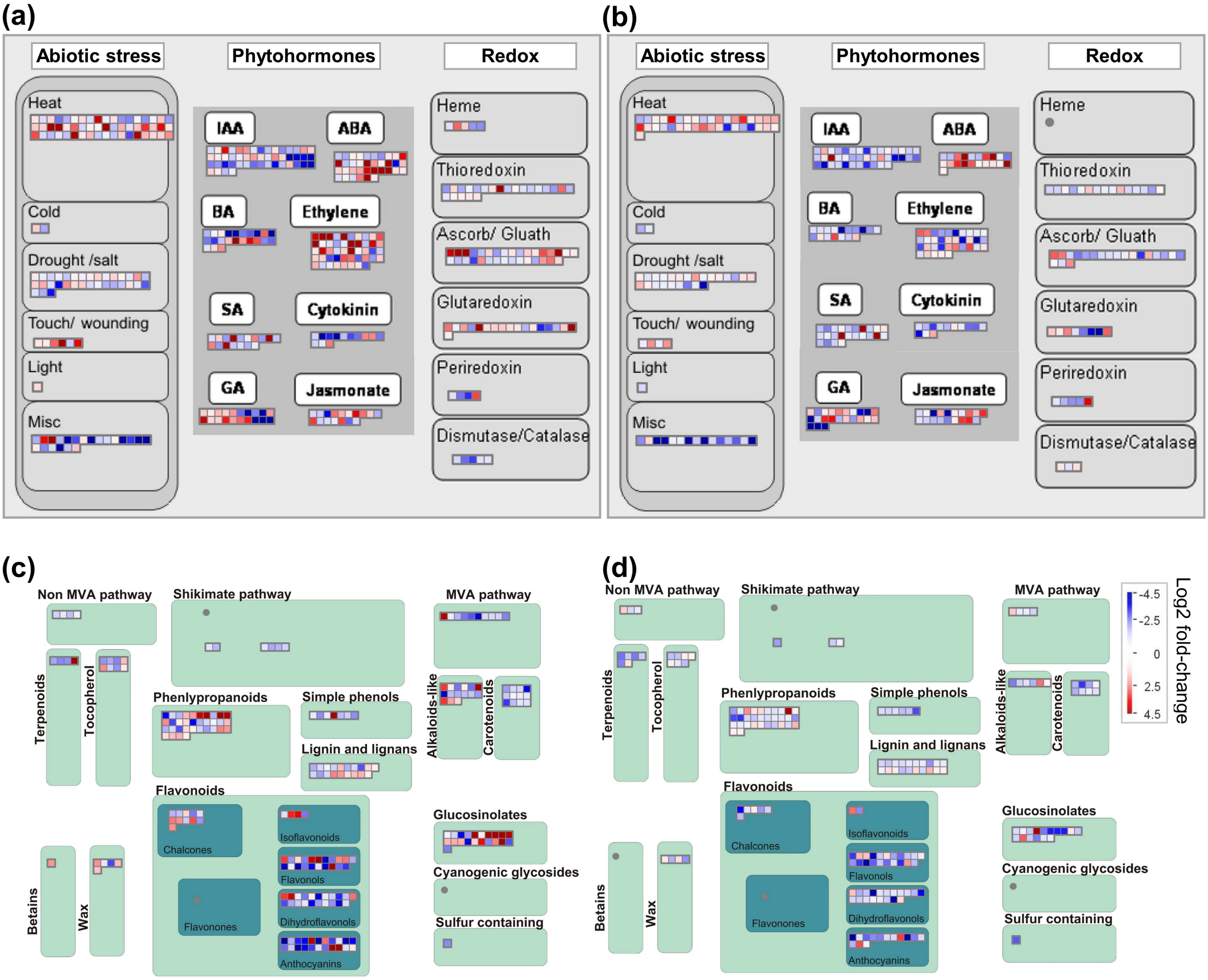

Figure S10. Transcriptional alterations in abiotic stress response, as well as phytohormone-, redox- and secondary metabolism-related pathways in 'sativa' (**a** and **c**) and 'falcata' (**b** and **d**) plants under control vs. drought conditions. Pathway analysis was conducted using MapMan, with blue boxes indicating down-regulated genes and red boxes denoting up-regulated genes.
