# Supplementary material for: Identification of Differential Drought Response Mechanisms in Medicago sativa subsp. sativa and falcata through Comparative Assessments at the Physiological, Biochemical, and Transcriptional Levels
Source: Plants (Basel). 2021 Oct 5;10(10):2107. doi: 10.3390/plants10102107 (PMC8539336; doi:10.3390/plants10102107)
Supplement: Supplementary file 1 [file plants-10-02107-s001.zip › Supplemental Figure 12 Mapman sativa vs falcata redox hormones (Aug 24 2021).pdf]

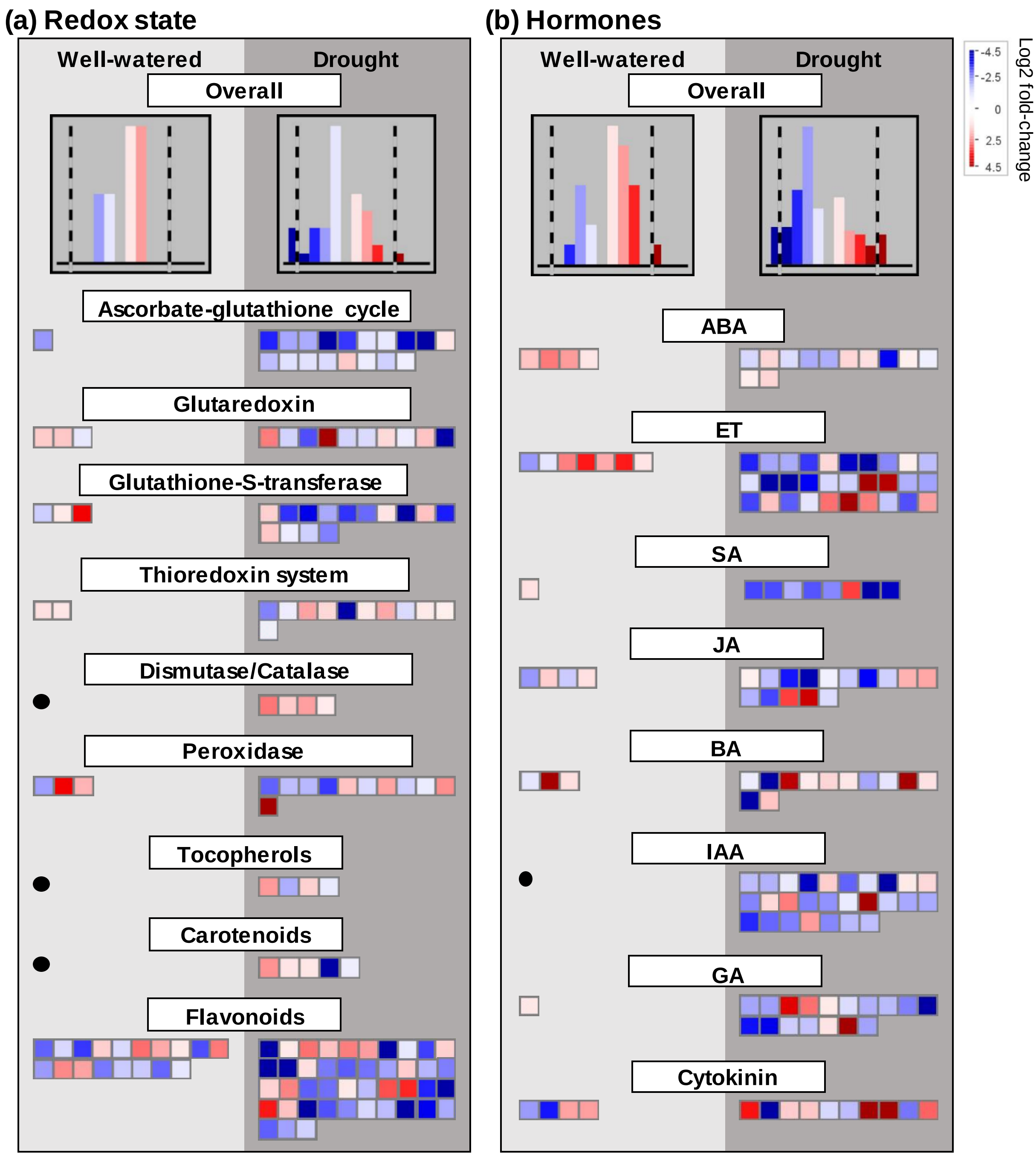

Figure S12. Transcriptional alterations in redox-related pathways, as well as hormone metabolism, between 'sativa' vs. 'falcata' under control and drought conditions. Pathway analysis was conducted using MapMan, with blue boxes indicating down-regulated genes and red boxes denoting up-regulated genes.
