# Supplementary material for: Identification of Differential Drought Response Mechanisms in Medicago sativa subsp. sativa and falcata through Comparative Assessments at the Physiological, Biochemical, and Transcriptional Levels
Source: Plants (Basel). 2021 Oct 5;10(10):2107. doi: 10.3390/plants10102107 (PMC8539336; doi:10.3390/plants10102107)
Supplement: Supplementary file 1 [file plants-10-02107-s001.zip › Supplemental Figure 13 Combined protective proteins heat map (Aug 24 2021).pdf]

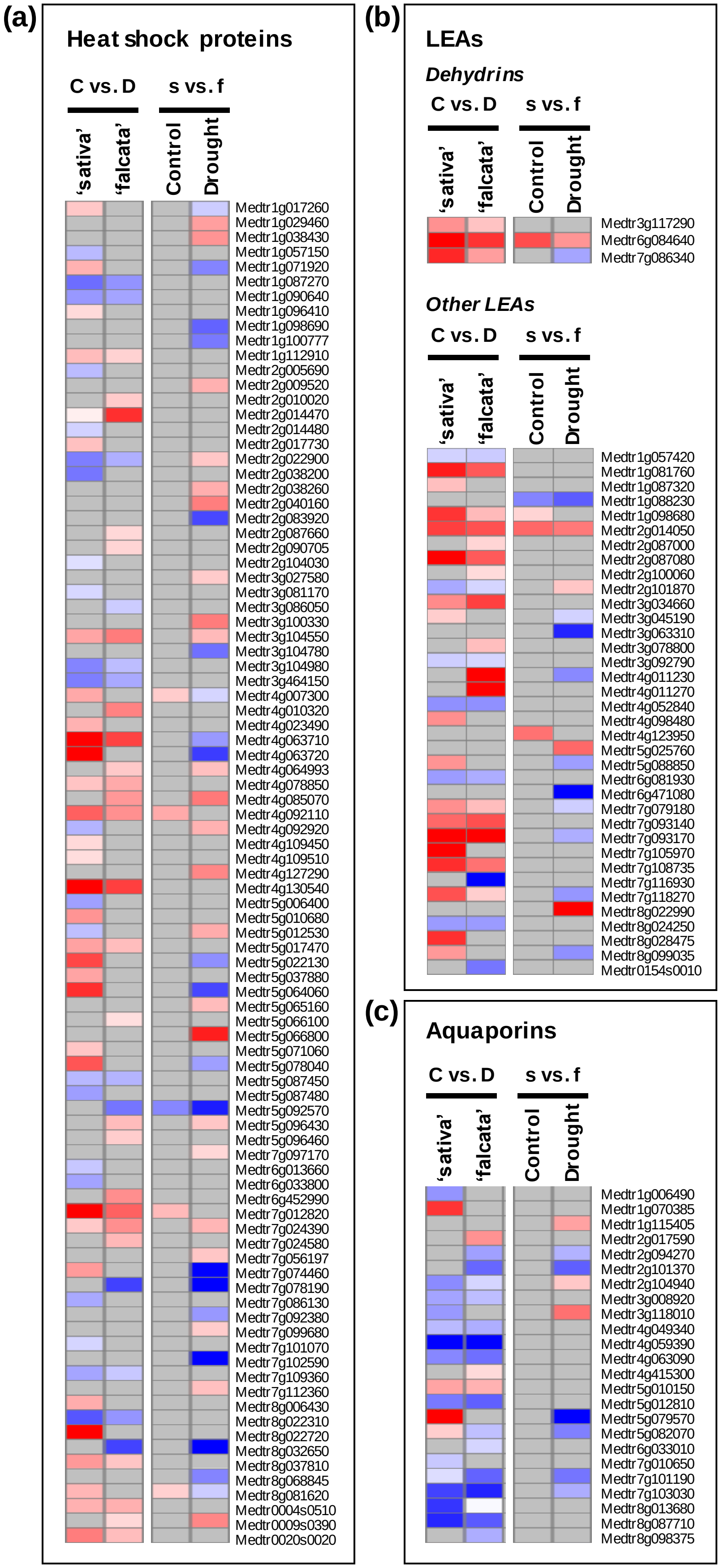

Figure S13. Differential expression of genes encoding heat shock factors, LEAs and aquaporins between conditions and genotypes. Blue boxes represent down-regulated genes and red boxes denote up-regulated genes, with the intensity of colour indicating the degree of log2 fold-change. C, control; D, drought; f, 'falcata'; s, 'sativa'.
