# Supplementary material for: Identification of Differential Drought Response Mechanisms in Medicago sativa subsp. sativa and falcata through Comparative Assessments at the Physiological, Biochemical, and Transcriptional Levels
Source: Plants (Basel). 2021 Oct 5;10(10):2107. doi: 10.3390/plants10102107 (PMC8539336; doi:10.3390/plants10102107)
Supplement: Supplementary file 1 [file plants-10-02107-s001.zip › Supplemental Figure 2 Proline carb and antioxidants (Dec 2 2020).pdf]

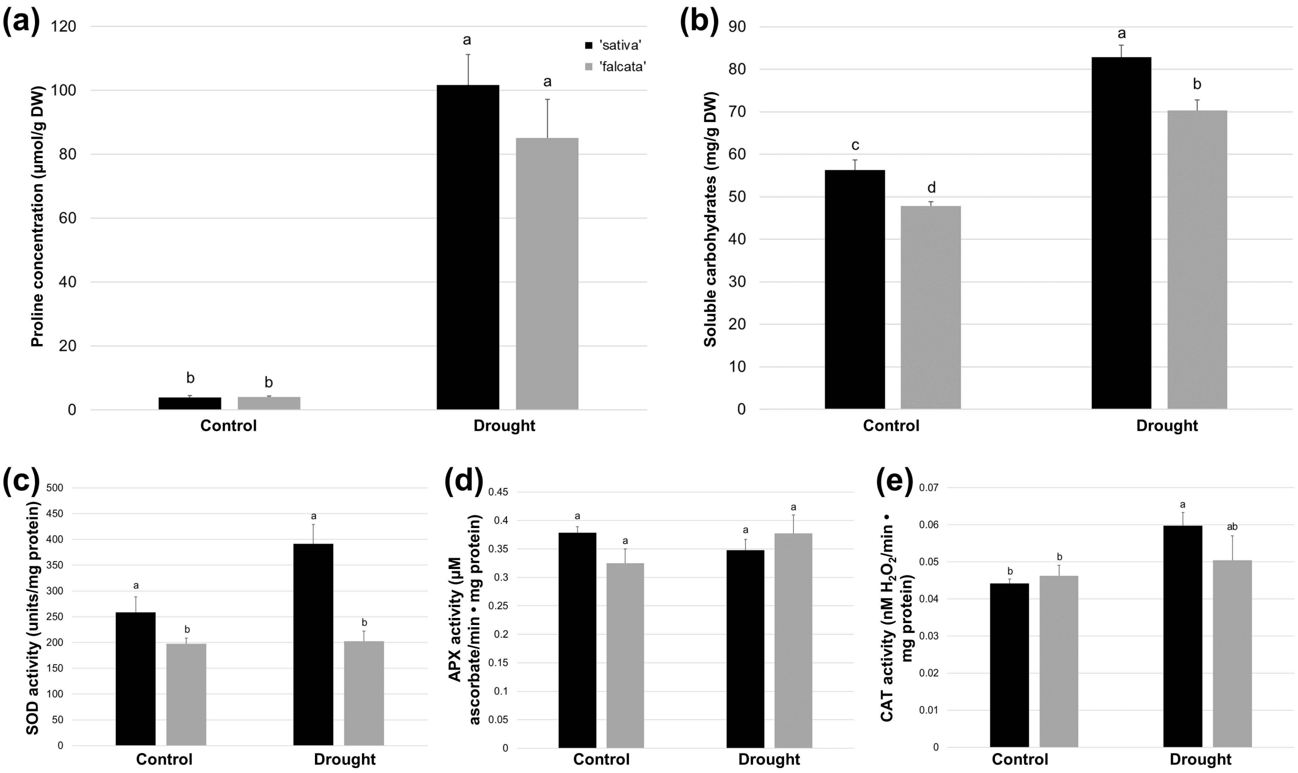

Figure S2. Biochemical and antioxidant response of 'sativa' and 'falcata' genotypes to drought stress conditions. All assessments were carried out on freeze dried first fully expanded leaves from 'sativa' (black) and 'falcata' (gray) plants under well-watered conditions (control) and when soil moisture content reached approximately 7% (drought). **(a)** Proline concentration in 'sativa' and 'falcata' leaves. Blocks in each graph consist of the mean value of 9-10 biological replicates derived from stem cuttings. **(b)** Concentration of soluble carbohydrates in 'sativa' and 'falcata' leaves. Blocks in each graph consist of the mean value of 9-10 biological replicates derived from stem cuttings. **(c-e)** Enzymatic antioxidant activity in 'sativa' and 'falcata' leaves. Blocks in each graph consist of the mean value of 9-11 biological replicates derived from stem cuttings. For all graphs, bars denote standard errors. Lowercase letters indicate statistically significant differences between groups in each graph ($p \leq 0.05$) as determined through the generation of mixed generalized models using the GLIMMIX procedure in SAS, and the subsequent use of Bonferroni's method to adjust for multiple comparisons.
