# Supplementary material for: Identification of Differential Drought Response Mechanisms in Medicago sativa subsp. sativa and falcata through Comparative Assessments at the Physiological, Biochemical, and Transcriptional Levels
Source: Plants (Basel). 2021 Oct 5;10(10):2107. doi: 10.3390/plants10102107 (PMC8539336; doi:10.3390/plants10102107)
Supplement: Supplementary file 1 [file plants-10-02107-s001.zip › Supplemental Figure 3 PCA analysis (June 15 2021).pdf]

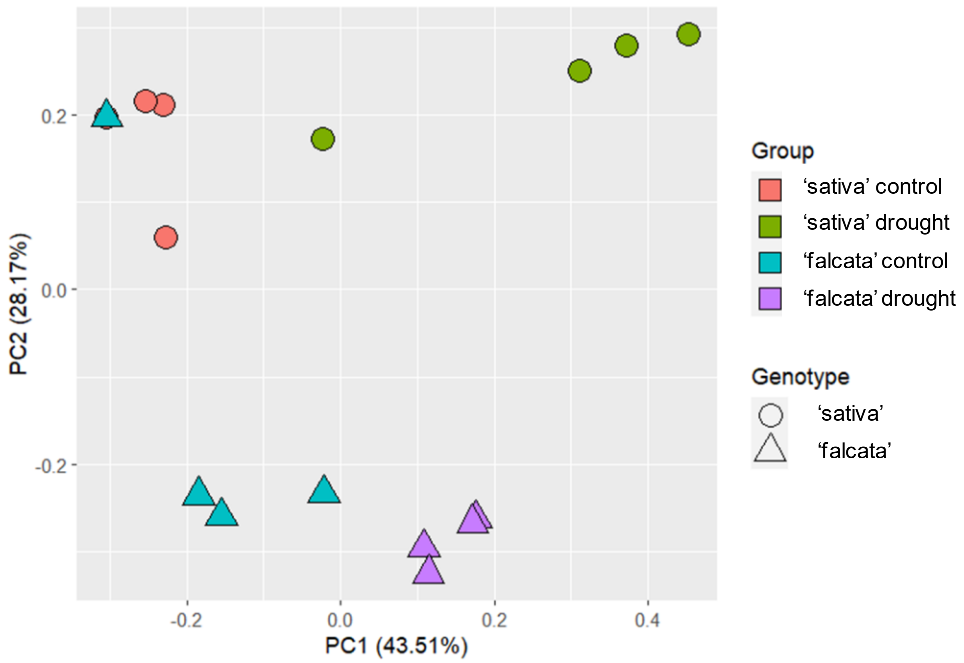

Figure S3. Principal component analysis of FPKM expression values. Four biological replicates of each genotype ('sativa' and 'falcata') under each treatment (control and drought) were assessed.
