# Supplementary material for: Identification of Differential Drought Response Mechanisms in Medicago sativa subsp. sativa and falcata through Comparative Assessments at the Physiological, Biochemical, and Transcriptional Levels
Source: Plants (Basel). 2021 Oct 5;10(10):2107. doi: 10.3390/plants10102107 (PMC8539336; doi:10.3390/plants10102107)
Supplement: Supplementary file 1 [file plants-10-02107-s001.zip › Supplemental Figure 4 Venn diagrams and qRT-PCR validation (Apr 29 2021).pdf]

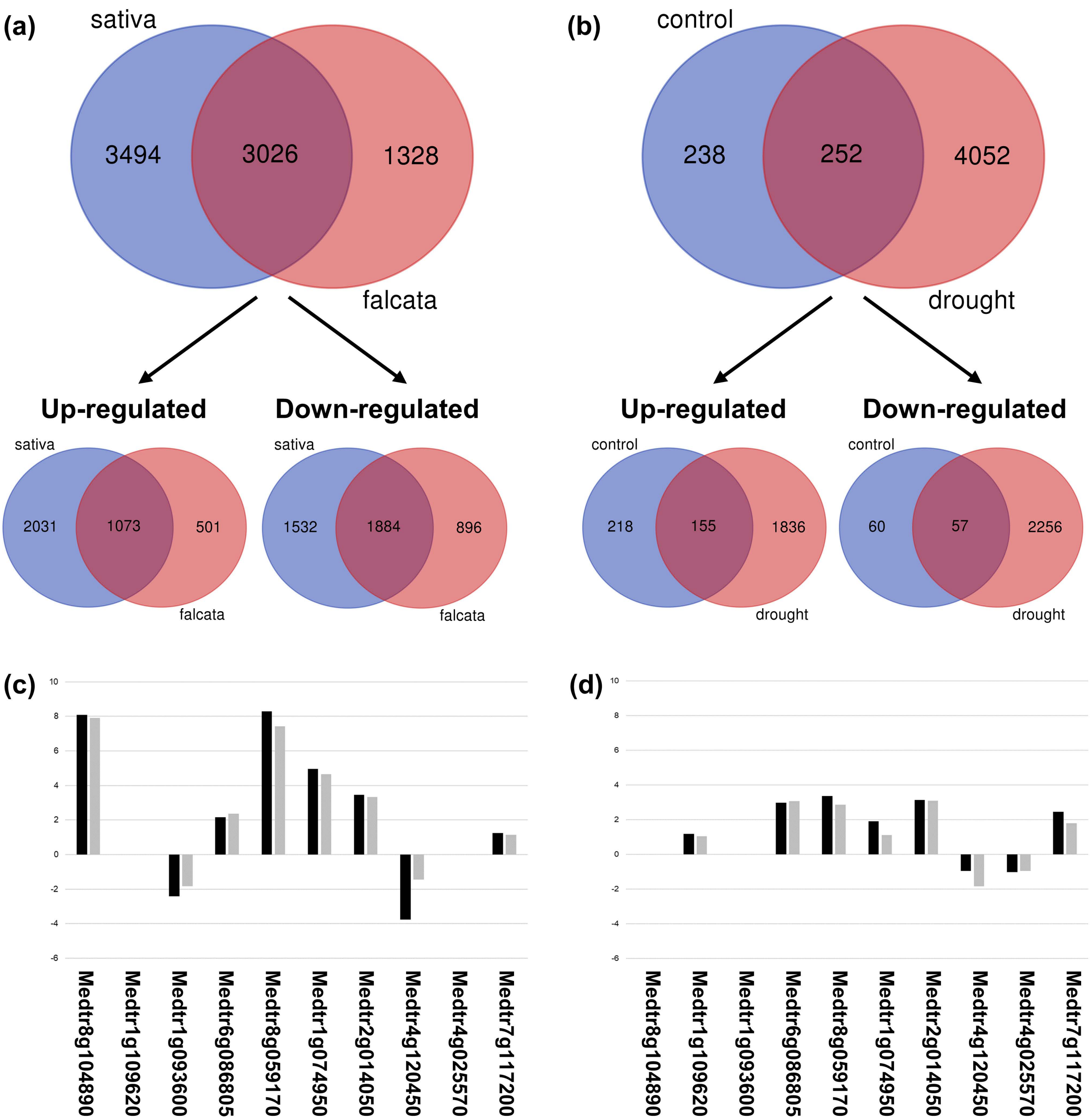

Figure S3. Identification of DEGs between genotypes and conditions and validation of RNA-Seq results. **(a)** Total, up-regulated and down-regulated DEGs with a p-value < 0.05 observed between control and drought conditions in 'sativa' (blue) and 'falcata' (pink) genotypes, with DEGs that are present in both genotypes shown in overlapping regions. **(b)** Total, up-regulated and down-regulated DEGs with a p-value < 0.05 observed between 'sativa' and 'falcata' under well-watered control (blue) and drought (pink) conditions, with DEGs that are present under both growth conditions shown in overlapping regions of the circles. **(c)** and **(d)** Correlation between RNA-Seq and qRT-PCR data in 'sativa' **(c)** and 'falcata' **(d)**. Bars denote log2-fold changes between control and drought conditions with RNA-Seq values displayed in black and qRT-PCR values shown in gray.
