# Supplementary material for: Identification of Differential Drought Response Mechanisms in Medicago sativa subsp. sativa and falcata through Comparative Assessments at the Physiological, Biochemical, and Transcriptional Levels
Source: Plants (Basel). 2021 Oct 5;10(10):2107. doi: 10.3390/plants10102107 (PMC8539336; doi:10.3390/plants10102107)
Supplement: Supplementary file 1 [file plants-10-02107-s001.zip › Supplemental Figure 5 SEACOMPARE up regulated biological process (Jan 8 2021).pdf]

				1		2	
**GO term**	**Description**	**1**	**2**	**p**	**Num**	**p**	**Num**
GO:0080090	Regulation of primary metabolic process			-	-	$8.8e^{-05}$	98
GO:0019222	Regulation of metabolic process			-	-	$8.8e^{-05}$	103
GO:0060255	Regulation of macromolecule metabolic process			-	-	$8.8e^{-05}$	100
GO:0009889	Regulation of biosynthetic process			-	-	$8.8e^{-05}$	98
GO:0006355	Regulation of transcription, DNA-dependent			-	-	$8.8e^{-05}$	97
GO:0010556	Regulation of macromolecule biosynthetic process			-	-	$8.8e^{-05}$	98
GO:0006350	Transcription			-	-	$8.8e^{-05}$	104
GO:0006351	Transcription, DNA-dependent			-	-	$8.8e^{-05}$	104
GO:0051171	Regulation of nitrogen compound metabolic process			-	-	$8.8e^{-05}$	98
GO:0032774	RNA biosynthetic process			-	-	$8.8e^{-05}$	104
GO:0031326	Regulation of cellular biosynthetic process			-	-	$8.8e^{-05}$	98
GO:0031323	Regulation of cellular metabolic process			-	-	$8.8e^{-05}$	101
GO:0045449	Regulation of transcription			-	-	$8.8e^{-05}$	97
GO:0010468	Regulation of gene expression			-	-	$8.8e^{-05}$	99
GO:0019219	Regulation of nucleobase, nucleoside, nucleotide, nucleic acid metabolic process			-	-	$8.8e^{-05}$	98
GO:0051252	Regulation of RNA metabolic process			-	-	$9.1e^{-05}$	97
GO:0016070	RNA metabolic process			-	-	$5.3e^{-04}$	115
GO:0006807	Nitrogen compound metabolic process			-	-	$8.0e^{-04}$	164
GO:0006457	Protein folding			-	-	$1.3e^{-03}$	21
GO:0065007	Biological regulation			-	-	$2.1e^{-03}$	140
GO:0006139	Nucleobase, nucleoside, nucleotide, nucleic acid metabolic process			-	-	$3.2e^{-03}$	139
GO:0042254	Ribosome biogenesis			-	-	$3.9e^{-03}$	9
GO:0022613	Ribonucleoprotein complex biogenesis			-	-	$3.9e^{-03}$	9
GO:0050789	Regulation of biological process			-	-	$5.1e^{-03}$	136
GO:0050794	Regulation of cellular process			-	-	$6.0e^{-03}$	134
GO:0044237	Cellular metabolic process			-	-	$9.1e^{-03}$	329
GO:0044262	Cellular carbohydrate metabolic process			-	-	$2.1e^{-02}$	23
GO:0034645	Cellular macromolecule biosynthetic process			-	-	$2.2e^{-02}$	124
GO:0009059	Macromolecule biosynthetic process			-	-	$2.2e^{-02}$	124
GO:0010467	Gene expression			-	-	$2.5e^{-02}$	128
GO:0044260	Cellular macromolecule metabolic process			-	-	$3.7e^{-02}$	260
GO:0009987	Cellular process			-	-	$3.7e^{-02}$	403
GO:0006470	Protein amino acid dephosporylation			-	-	$4.8e^{-02}$	11

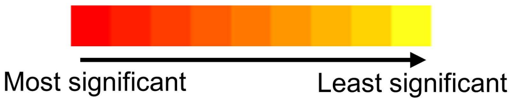

Figure S5. SEACOMPARE analysis of up-regulated DEGs observed in 'sativa' control vs. drought (1) and 'falcata' control vs. drought (2), respectively, in the biological process GO grouping. Analysis was carried out using the AgriGO v2.0 program by cross comparing SEA enrichment results for each. P, adjusted *p*-value; Num, number of DEGs within GO group.
