# Supplementary material for: Identification of Differential Drought Response Mechanisms in Medicago sativa subsp. sativa and falcata through Comparative Assessments at the Physiological, Biochemical, and Transcriptional Levels
Source: Plants (Basel). 2021 Oct 5;10(10):2107. doi: 10.3390/plants10102107 (PMC8539336; doi:10.3390/plants10102107)
Supplement: Supplementary file 1 [file plants-10-02107-s001.zip › Supplemental Figure 6 SEACOMPARE up regulated molecular function (Jan 8 2021).pdf]

				1		2	
**GO term**	**Description**	**1**	**2**	**p**	**Num**	**p**	**Num**
GO:0005509	Calcium ion binding			$4.3e^{-05}$	63	$8.7e^{-05}$	39
GO:0043565	Sequence-specific DNA binding			$1.0e^{-04}$	74	$1.9e^{-03}$	42
GO:0042578	Phosphoric ester hydrolase activity			$2.8e^{-04}$	47	$4.0e^{-02}$	24
GO:0016791	Phosphatase activity			$5.3e^{-03}$	36	-	-
GO:0004842	Ubiquitin-protein ligase activity			$1.4e^{-02}$	26	-	-
GO:0003700	Transcription factor activity			$1.4e^{-02}$	97	$8.7e^{-05}$	67
GO:0019787	Small conjugating protein ligase activity			$1.4e^{-02}$	26	-	-
GO:0046872	Metal ion binding			$1.8e^{-02}$	341	0.004	334
GO:0043167	Ion binding			$1.8e^{-02}$	342	0.004	334
GO:0043169	Cation binding			$1.8e^{-08}$	342	-	-
GO:0004722	Protein serine/threonine phosphatase activity			$1.9e^{-02}$	15	-	-
GO:0004674	Protein serine/threonine kinase activity			$3.0e^{-02}$	151	-	-
GO:0004721	Phosphoprotein phosphatase activity			$3.4e^{-02}$	21	-	-
GO:0030528	Transcription factor regulator activity			-	-	$1.9e^{-03}$	68
GO:0003677	DNA binding			-	-	$3.6e^{-02}$	133
GO:0051082	Unfolded protein binding			-	-	$3.6e^{-02}$	12

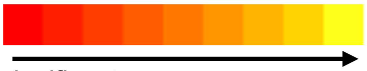

Figure S6. SEACOMPARE analysis of up-regulated DEGs observed in 'sativa' control vs. drought (1) and 'falcata' control vs. drought (2), respectively, in the molecular function GO grouping. Analysis was carried out using the AgriGO v2.0 program by cross comparing SEA enrichment results for each. P, adjusted *p*-value; Num, number of DEGs within GO term.
