# Supplementary material for: Identification of Differential Drought Response Mechanisms in Medicago sativa subsp. sativa and falcata through Comparative Assessments at the Physiological, Biochemical, and Transcriptional Levels
Source: Plants (Basel). 2021 Oct 5;10(10):2107. doi: 10.3390/plants10102107 (PMC8539336; doi:10.3390/plants10102107)
Supplement: Supplementary file 1 [file plants-10-02107-s001.zip › Supplemental Figure 7 SEACOMPARE down regulated biological process (Jan 8 2021).pdf]

				1		2	
**GO term**	**Description**	**1**	**2**	**p**	**Num**	**p**	**Num**
GO:0008152	Metabolic process			$3.3e^{-28}$	1153	$8.3e^{-19}$	903
GO:0006412	Translation			$2.2e^{-24}$	154	$1.7e^{-03}$	72
GO:0009058	Biooynthetic process			$1.1e^{-23}$	437	$1.5e^{-03}$	264
GO:0044249	Cellular biosynthetic process			$1.3e^{-23}$	429	$1.7e^{-03}$	258
GO:0034641	Cellular nitrogen compound metabolic process			$4.2e^{-23}$	138	$2.2e^{-07}$	81
GO:0044281	Small molecule metabolic process			$6.7e^{-22}$	232	$5.5e^{-09}$	157
GO:0044238	Primary metabolic process			$9.1e^{-21}$	874	$8.7e^{-06}$	627
GO:0043436	Oxoacid metabolic process			$3.8e^{-19}$	128	$1.2e^{-06}$	78
GO:0042180	Cellular ketone metabolic process			$3.8e^{-19}$	129	$1.2e^{-06}$	79
GO:0019752	Carboxylic acid metabolic process			$3.8e^{-19}$	128	$1.2e^{-06}$	78
GO:0006082	Organic acid metabolic process			$3.9e^{-19}$	128	$1.2e^{-06}$	78
GO:0044237	Cellular metabolic process			$7.9e^{-19}$	785	$2.8e^{-03}$	539
GO:0044271	Cellular nitrogen compound biosynthetic process			$3.8e^{-18}$	87	$1.5e^{-04}$	45
GO:0044283	Small molecule biosynthetic process			$2.9e^{-17}$	100	$6.7e^{-05}$	56
GO:0006520	Cellular amino acid metabolic process			$6.5e^{-15}$	94	$3.7e^{-04}$	53
GO:0006519	Cellular amino acid and derivative metabolic process			$1.3e^{-14}$	107	$1.4e^{-04}$	64
GO:0044106	Cellular amine metabolic process			$1.3e^{-14}$	98	$1.3e^{-03}$	54
GO:0046394	Carboxylic acid biosynthetic process			$1.6e^{-13}$	69	$5.5e^{-04}$	38
GO:0016053	Organic acid biosynthetic process			$1.6e^{-13}$	69	$5.5e^{-04}$	38
GO:0009308	Amine metabolic process			$2.7e^{-13}$	101	$2.4e^{-03}$	57
GO:0009987	Cellular process			$6.8e^{-13}$	920	-	-
GO:0008652	Cellular amino acid biosynthetic process			$9.6e^{-11}$	51	$4.1e^{-02}$	24
GO:0018130	Heterocycle biosynthetic process			$6.8e^{-10}$	38	$3.7e^{-04}$	23
GO:0046483	Heterocycle metabolic process			$1.3e^{-09}$	83	$5.0e^{-05}$	59
GO:0009309	Amine biosynthetic process			$1.4e^{-09}$	52	-	-
GO:0005975	Carbohydrate metabolic process			$3.7e^{-08}$	140	$6.7e^{-08}$	120
GO:0006629	Lipid metabolic process			$4.2e^{-07}$	60	$5.5e^{-04}$	43
GO:0006725	Cellular aromatic compound metabolic process			$4.4e^{-06}$	47	$9.5e^{-03}$	31
GO:0034645	Cellular macromolecule biosynthetic process			$8.6e^{-06}$	268	-	-
GO:0009059	Macromolecule biosynthetic process			$9.4e^{-06}$	268	-	-
GO:0010457	Gene expression			$1.1e^{-05}$	277	-	-
GO:0044255	Cellular lipid metabolic process			$1.8e^{-05}$	47	$2.1e^{-02}$	31
GO:0033014	Tetrapyrrole biosynthetic process			$1.9e^{-05}$	17	$5.4e^{-05}$	15
GO:0033013	Tetrapyrrole metabolic process			$1.9e^{-05}$	17	$5.4e^{-05}$	15
GO:0006807	Nitrogen compound metabolic process			$4.6e^{-05}$	324	-	-
GO:0008610	Lipid biosynthetic process			$1.0e^{-04}$	33	-	-
GO:0043648	Dicarboxylic acid metabolic process			$1.9e^{-04}$	22	-	-
GO:0019538	Protein metabolic process			$2.6e^{-04}$	407	-	-
GO:0006563	L-serine metabolic process			$2.7e^{-04}$	15	-	-

				1		2	
**GO term**	**Description**	**1**	**2**	**p**	**Num**	**p**	**Num**
GO:0061188	Cofactor biosynthetic process			$2.9e^{-04}$	30	$4.9e^{-03}$	23
GO:0009069	Serine family amino acid metabolic process			$3.0e^{-04}$	17	-	-
GO:0034660	ncRNA metabolic process			$5.0e^{-04}$	30	-	-
GO:0051186	Cofactor metabolic process			$5.0e^{-04}$	39	$3.9e^{-03}$	31
GO:0044267	Cellular protein metabolic process			$8.0e^{-04}$	349	-	-
GO:0055086	Nucleobase,nucleoside and nucleotide metabolic process			$1.2e^{-03}$	54	-	-
GO:0006399	tRNA metabolic process			$1.6e^{-03}$	26	-	-
GO:0043039	tRNA aminoacylation			$2.0e^{-03}$	21	-	-
GO:0006418	tRNA aminoacylation for protein translation			$2.0e^{-03}$	21	-	-
GO:0043038	Amino acid activation			$2.0e^{-03}$	21	-	-
GO:0006790	Sulfur metabolic process			$2.2e^{-03}$	25	$2.6e^{-02}$	19
GO:0009070	Serine family amino acid biosynthetic process			$2.2e^{-03}$	12	-	-
GO:0044262	Cellular carbohydrate metabolic process			$2.6e^{-03}$	43	$1.6e^{-03}$	38
GO:0006778	Porphyrin metabolic process			$3.0e^{-03}$	11	$3.2e^{-03}$	10
GO:0006779	Porphyrin biosynthetic process			$3.0e^{-03}$	11	$3.2e^{-03}$	10
GO:0044272	Sulfur compound biosynthetic process			$3.2e^{-03}$	17	-	-
GO:0034404	Nucleobase, nucleoside and nucleotide biosynthetic process			$8.0e^{-03}$	10	-	-
GO:0034654	Nucleobase, nucleoside, nucleotide and nucleic acid biosynthetic process			$8.0e^{-03}$	10	-	-
GO:0006457	Protein folding			$1.0e^{-02}$	31	-	-
GO:0042434	Indole derivative metabolic process			$1.0e^{-02}$	8	-	-
GO:0006586	Indolalkylamine metabolic process			$1.0e^{-02}$	8	-	-
GO:0042430	Indole and derivative metabolic process			$1.0e^{-02}$	8	-	-
GO:0006568	Tryptophan metabolic process			$1.0e^{-02}$	8	-	-
GO:0044282	Small molecule catabolic process			$1.0e^{-02}$	27	$1.6e^{-02}$	23
GO:0000097	Sulful amino acid biosynthetic process			$1.0e^{-02}$	12	-	-
GO:0009119	Ribonucleoside metabolic process			$1.0e^{-02}$	14	-	-
GO:0032787	Monocarboxylic acid metabolic process			$1.1e^{-02}$	29	-	-
GO:0019748	Secondary metabolic process			$1.2e^{-02}$	19	-	-
GO:0000162	Tryptophan biosynthetic process			$1.3e^{-02}$	6	-	-
GO:0042435	Indole derivative biosynthetic process			$1.3e^{-02}$	6	-	-
GO:0046219	Indolalkylamine biosynthetic process			$1.3e^{-02}$	6	-	-
GO:0009116	Nucleoside metabolic process			$1.4e^{-02}$	14	-	-
GO:0043170	Macromolecule metabolic process			$1.5e^{-02}$	577	-	-
GO:0006575	Cellular amino acid derivative metabolic process			$1.7e^{-02}$	27	-	-
GO:0006633	Fatty acid biosynthetic process			$1.7e^{-02}$	16	-	-
GO:0006753	Nucleoside phosphate metabolic process			$2.1e^{-02}$	44	-	-
GO:0006066	Alcohol metabolic process			$2.1e^{-02}$	34	-	-
GO:0009117	Nucleotide metabolic process			$2.1e^{-02}$	44	-	-
GO:0000096	Sulfur amino acid metabolic process			$2.3e^{-02}$	16	-	-

				1		2	
**GO term**	**Description**	**1**	**2**	**p**	**Num**	**p**	**Num**
GO:0008299	Isoprenoid biosynthetic process			$2.4e^{-02}$	13	-	-
GO:0006720	Isoprenoid metabolic process			$2.4e^{-02}$	13	-	-
GO:0042440	Pigment metabolic process			$2.9e^{-02}$	10	-	-
GO:0046148	Pigment biosynthetic process			$2.9e^{-02}$	10	-	-
GO:0009110	Vitamin biosynthetic process			$2.9e^{-02}$	11	-	-
GO:0006766	Vitamin metabolic process			$2.9e^{-02}$	11	-	-
GO:0019344	Cysteine biosynthetic process			$2.9e^{-02}$	8	-	-
GO:0005996	Monosaccharide metabolic process			$2.9e^{-02}$	28	$2.1e^{-02}$	25
GO:0006535	Cysteine biosynthetic process from serine			$2.9e^{-02}$	8	-	-
GO:0019318	Hexose metabolic process			$3.0e^{-02}$	24	-	-
GO:0006006	Glucose metabolic process			$3.8e^{-02}$	21	-	-
GO:0044260	Cellular macromolecule metabolic process			$3.9e^{-02}$	515	-	-
GO:0006800	Oxygen and reactive oxygen species metabolic process			$4.0e^{-02}$	6	-	-
GO:0006801	Superoxide metabolic process			$4.0e^{-02}$	6	-	-
GO:0071669	Plant-type cell wall organization or biogenesis			$4.0e^{-02}$	11	-	-
GO:0009664	Plant-type cell wall organization			$4.0e^{-02}$	11	-	-
GO:0019438	Aromatic compound biosynthetic process			$4.0e^{-02}$	19	-	-
GO:0046112	Nucleobase biosynthetic process			$4.0e^{-02}$	7	-	-
GO:0071555	Cell wall organization			$4.0e^{-02}$	11	-	-
GO:0009112	Nucleobase metabolic process			$4.6e^{-02}$	8	-	-
GO:0009081	Branched chain family amino acid metabolic process			$4.6e^{-02}$	8	-	-
GO:0006534	Cysteine metabolic process			$4.6e^{-02}$	8	-	-
GO:0044264	Cellular polysaccharide metabolic process			$4.6e^{-02}$	9	-	-
GO:0033692	Cellular polysaccharide biosynthetic process			$4.6e^{-02}$	9	-	-
GO:0015979	Photosynthesis			-	-	$3.2e^{-03}$	19
GO:0008272	Sulfate transport			-	-	$1.1e^{-02}$	8
GO:0055114	Oxidation reduction			-	-	$2.1e^{-02}$	167
GO:0015698	Inorganic anion transport			-	-	$3.9e^{-02}$	11

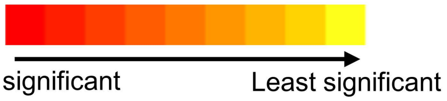

Figure S7. SEACOMPARE analysis of down-regulated DEGs observed in 'sativa' control vs. drought (1) and 'falcata' control vs. drought (2), respectively, in the biological process GO grouping. Analysis was carried out using the AgriGO v2.0 program by cross comparing SEA enrichment results for each. P, adjusted *p*-value; Num, number of DEGs within GO term.
