# Supplementary material for: Identification of Differential Drought Response Mechanisms in Medicago sativa subsp. sativa and falcata through Comparative Assessments at the Physiological, Biochemical, and Transcriptional Levels
Source: Plants (Basel). 2021 Oct 5;10(10):2107. doi: 10.3390/plants10102107 (PMC8539336; doi:10.3390/plants10102107)
Supplement: Supplementary file 1 [file plants-10-02107-s001.zip › Supplemental Figure 8 SEACOMPARE down regulated molecular function (Jan 8 2021).pdf]

				1		2	
**GO term**	**Description**	**1**	**2**	**p**	**Num**	**p**	**Num**
GO:0003735	Structural constituent of ribosome			$2.5e^{-20}$	117	-	-
GO:0005198	Structural molecule activity			$9.8e^{-19}$	122	-	-
GO:0003824	Catalytic activity			$2.0e^{-13}$	1069	$1.3e^{-13}$	874
GO:0016829	Lyase activity			$8.6e^{-06}$	61	$4.4e^{-03}$	44
GO:0016757	Transferase activity, transferring glycosyl groups			$3.0e^{-04}$	54	$2.9e^{-04}$	47
GO:0016830	Carbon-carbon lyase activity			$2.2e^{-03}$	26	-	-
GO:0016763	Transferase activity, transferring pentosyl groups			$6.1e^{-03}$	13	-	-
GO:0016875	Ligase activity, forming carbon-oxygen bonds			$6.1e^{-03}$	21	-	-
GO:0004812	Aminoacyl-tRNA ligase activity			$6.1e^{-03}$	21	-	-
GO:0016876	Ligase activity, forming aminoacyl-tRNA and related compounds			$6.1e^{-03}$	21	-	-
GO:0000287	Magnesium ion binding			$6.8e^{-03}$	25	-	-
GO:0048037	Cofactor binding			$1.9e^{-02}$	67	-	-
GO:0004553	Hydrolase activity, hydrolyzing O-glycosyl compounds			$2.9e^{-02}$	65	$2.6e^{-02}$	56
GO:0016861	Intramolecular oxidoreductase activity, interconverting aldoses and ketoses			$3.0e^{-02}$	7	-	-
GO:0016758	Transferase activity, transferring hexosyl groups			$3.0e^{-02}$	41	$2.6e^{-03}$	40
GO:0016616	Oxidoreductase activity, acting on the CH-OH group of donors, NAD or NADP as acceptor			$3.8e^{-02}$	26	-	-
GO:0016798	Hydrolase activity, acting on glycosyl bonds			$3.9e^{-02}$	65	$2.9e^{-02}$	56
GO:0015116	Sulfate transmembrane transporter activity			-	-	$2.9e^{-02}$	8
GO:0008271	Secondary active sulfate transmembrane transporter activity			-	-	$2.9e^{-02}$	8
GO:0004252	Serine-type endopeptidase activity			-	-	$3.3e^{-02}$	28
GO:0030246	Carbohydrate binding			-	-	$3.5e^{-02}$	18
GO:0008236	Serine-type peptidase activity			-	-	$3.5e^{-02}$	37
GO:0017171	Serine hydrolase activity			-	-	$3.5e^{-02}$	37
GO:0005215	Transporter activity			-	-	$4.4e^{-02}$	91
GO:0042578	Phosphoric ester hydrolase activity			-	-	$4.9e^{-02}$	34

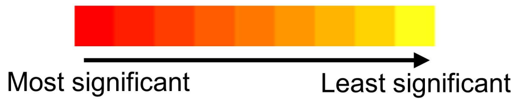

Figure S8. SEACOMPARE analysis of down-regulated DEGs observed in ‘sativa’ control vs. drought (1) and ‘falcata’ control vs. drought (2), respectively, in the molecular function GO grouping. Analysis was carried out using the AgriGO v2.0 program by cross comparing SEA enrichment results for each. P, adjusted *p*-value; Num, number of DEGs within GO term.
