# Supplementary material for: Identification of Differential Drought Response Mechanisms in Medicago sativa subsp. sativa and falcata through Comparative Assessments at the Physiological, Biochemical, and Transcriptional Levels
Source: Plants (Basel). 2021 Oct 5;10(10):2107. doi: 10.3390/plants10102107 (PMC8539336; doi:10.3390/plants10102107)
Supplement: Supplementary file 1 [file plants-10-02107-s001.zip › Supplemental Figure 9 SEACOMPARE down regulated cellular compartment heat map table (Dec 18 20).pdf]

				1		2	
**GO term**	**Description**	**1**	**2**	**p**	**Num**	**p**	**Num**
GO:0005737	Cytoplasm			$8.6e^{-25}$	214	0.0066	105
GO:0005840	Ribosome			$2.6e^{-21}$	117	0.032	48
GO:0030529	Ribonucleoprotein complex			$1.4e^{-20}$	120	-	-
GO:0043232	Intracellular non-membrane-bounded organelle			$1.4e^{-14}$	151	-	-
GO:0043228	Non-membrane-bounded organelle			$1.4e^{-14}$	151	-	-
GO:0044444	Cytoplasmic part			$2.5e^{-14}$	138	-	-
GO:0044424	Intracellular part			$7.6e^{-09}$	294	-	-
GO:0044464	Cell part			$1.8e^{-08}$	452	0.004	334
GO:0005623	Cell			$1.8e^{-08}$	452	0.004	334
GO:0043229	Macromolecular complex			$1.9e^{-08}$	179	-	-
GO:0043229	Intracellular			$3.1e^{-07}$	331	-	-
GO:0043226	Intracellular organelle			0.00016	196	-	-
GO:0033279	Organelle			0.00016	196	-	-
GO:0016020	Ribosomal subunit			0.0019	23	-	-
GO:0009521	Membrane			-	-	0.004	141
GO:0009521	Photosystem			-	-	0.0065	15
GO:0009579	Thylakoid			-	-	0.009	16
GO:0034357	Photosynthetic membrane			-	-	0.014	15
GO:0009522	Photosystem I			-	-	0.033	9

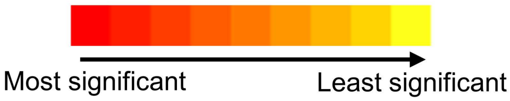

Figure S9. SEACOMPARE analysis of down-regulated DEGs observed in 'sativa' control vs. drought (1) and 'falcata' control vs. drought (2), respectively, in the cellular compartment GO grouping. Analysis was carried out using the AgriGO v2.0 program by cross comparing SEA enrichment results for each. P, adjusted *p*-value; Num, number of DEGs within GO term.
