# Supplementary material for: Identification of Differential Drought Response Mechanisms in Medicago sativa subsp. sativa and falcata through Comparative Assessments at the Physiological, Biochemical, and Transcriptional Levels
Source: Plants (Basel). 2021 Oct 5;10(10):2107. doi: 10.3390/plants10102107 (PMC8539336; doi:10.3390/plants10102107)
Supplement: Supplementary file 1 [file plants-10-02107-s001.zip › Supplemental Tables (June 11 2021).pdf]

Table S1. RNA-Seq read and alignment data for 'sativa' and 'falcata' under well-watered (control) and drought conditions

	# Bases	# Reads	Total number of transcripts
sC-2	16,159,747,702	79,998,751	35,143
sC-3	17,716,160,126	87,703,763	35,820
sC-4	19,263,496,690	95,363,845	37,411
sC-5	18,326,842,688	90,726,944	35,820
sD-1	16,550,608,208	81,933,704	37,386
sD-2	36,006,938,340	178,252,170	42,042
sD-4	16,134,470,028	79,873,614	37,069
sD-5	16,330,875,234	80,845,917	36,100
fC-1	19,258,345,488	95,338,344	37,530
fC-2	20,139,635,532	99,701,166	38,307
fC-3	22,815,279,860	112,946,930	35,820
fC-5	16,322,648,582	80,805,191	35,205
fD-1	21,876,937,946	108,301,673	39,714
fD-2	18,289,591,666	90,542,533	38,714
fD-3	18,477,974,038	91,475,119	37,801
fD-5	32,108,678,104	158,953,852	35,936

fC, falcata control (well-watered); fD, falcata drought; sC, sativa control (well-watered); sD, sativa drought

Table S2. Primers used for qRT-PCR validation of RNA-Seq results

Gene ID	Description	Primer sequences (5' – 3')	Amplicon length (bp)
Medtr8g104890	Plant cadmium resistance protein	F: CATGGAAATGTGGCACAAGG<br>R: ATGGATTCTCCCGACTTCAAC	94
Medtr1g109620	Trehalose-6-phosphate synthase domain protein	F: GGGTATTGTTGCAGAACGTATTC<br>R: GATAGAGATGCCCTTGCACTT	135
Medtr1g093600	AP2/ERF and B3 domain transcription factor	F: TGTCGGTGGTGAGGTTGATA<br>R: AAAGCCGAACCATCTGAACC	92
Medtr6g086805	Heat shock transcription factor	F: GTGAAGTTCCACGGATGCC<br>R: TTTCCGCCGCCATTTCATTG	120
Medtr8g059170	NAC transcription factor-like protein	F: GGGATGCTGAATTACGGTTGTA<br>R: GTCGGTGACCCGGTTAG	138
Medtr1g074950	Sieve element occlusion protein	F: GGCTGAATTTGAGAAATGGAAAGA<br>R: GATGAGTGACACGAGCAACA	95
Medtr2g014050	Late embryogenesis abundant domain protein	F: GAAGAATGCAGGAGATCAGGC<br>R: AATATCCCTTACTCCTTCCCAGC	90
Medtr4g120450	DnaJ heat shock amino-terminal domain protein	F: AGACTTCTAGTATGCTGCACAA<br>R: GTAAACATACTCCTTGATTTGACCTC	85
Medtr4g025570	Flavonoid O-methyltransferase-like protein	F: GGTGATGAAGAGTGCATTCAAA<br>R: GTTTCCTCCTTCATCAATCACTG	105
Medtr7g117200	WRKY family transcription factor	F: AGCTCCAACTTCAGTCACCA<br>R: CAGGAGTATTGAGAGGGAAATCA	95
Medtr2g028670	Actin depolymerizing factor	F: GCATCTGGTATGGCAGTCC<br>R: GCACTCATCAGCAGGAAGG	183
