# Supplementary material for: Identification of Differential Drought Response Mechanisms in Medicago sativa subsp. sativa and falcata through Comparative Assessments at the Physiological, Biochemical, and Transcriptional Levels
Source: Plants (Basel). 2021 Oct 5;10(10):2107. doi: 10.3390/plants10102107 (PMC8539336; doi:10.3390/plants10102107)
Supplement: Supplementary file 1 [file Supplemental Figure 11 Mapman transcription factors sativa and falcata control vs drought (Jan 25 2021).pdf › Supplemental Figure 11 Mapman transcription factors sativa and falcata control vs drought (Jan 25 2021).pdf]

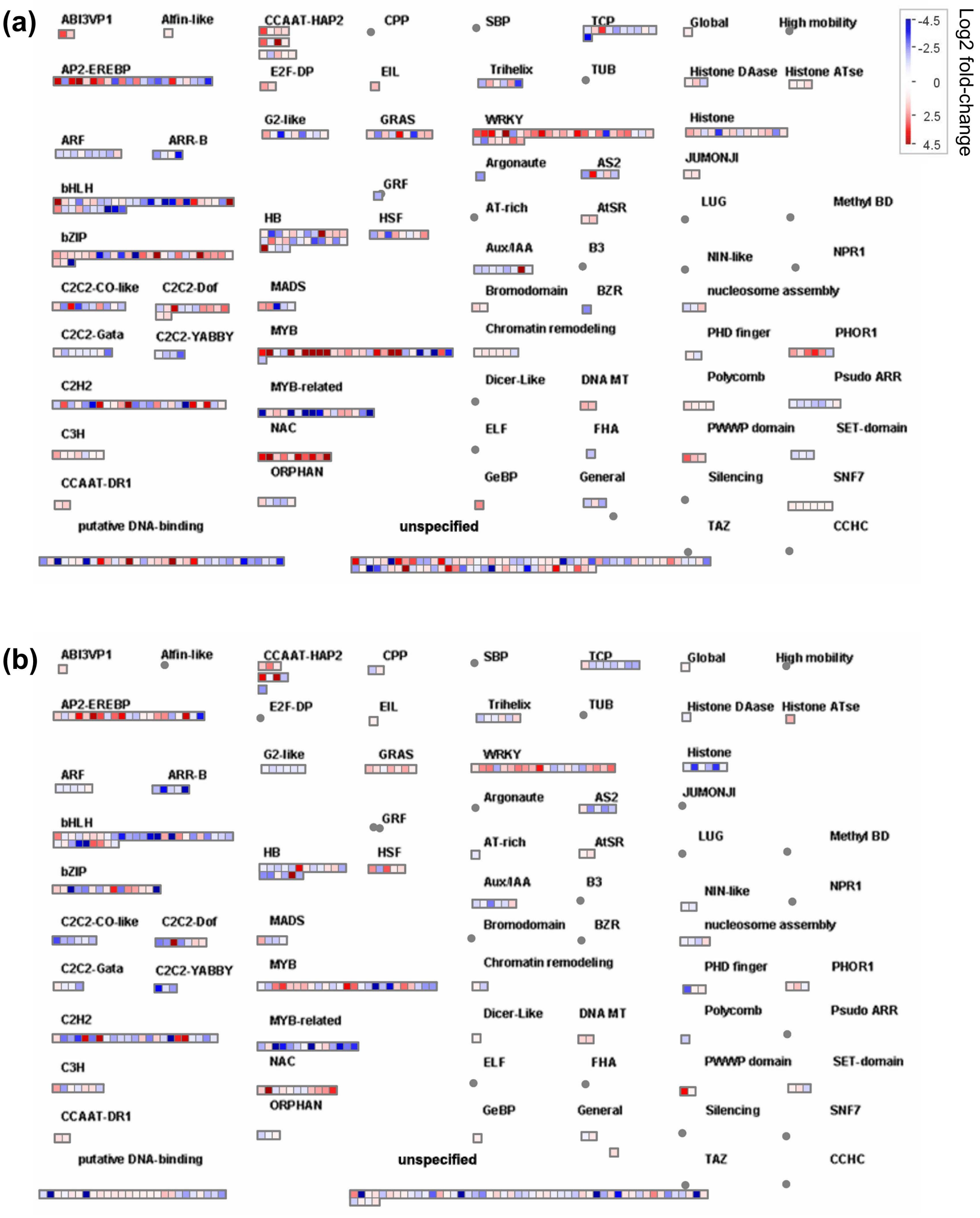

Figure S11. Transcriptional alterations in genes involved in transcriptional regulation in 'sativa' (**a**) and 'falcata' (**b**) plants under control vs. drought conditions. Pathway analysis was conducted using MapMan, with blue boxes indicating down-regulated genes and red boxes denoting up-regulated genes.
